# Histamine H3 receptors aggravate cerebral ischaemic injury by histamine-independent mechanisms

**DOI:** 10.1038/ncomms4334

**Published:** 2014-02-25

**Authors:** Haijing Yan, Xiangnan Zhang, Weiwei Hu, Jing Ma, Weiwei Hou, Xingzhou Zhang, Xiaofen Wang, Jieqiong Gao, Yao Shen, Jianxin Lv, Hiroshi Ohtsu, Feng Han, Guanghui Wang, Zhong Chen

**Affiliations:** 1 Department of Pharmacology, Key Laboratory of Medical Neurobiology of the Ministry of Health of China, Zhejiang Province Key Laboratory of Neurobiology, College of Pharmaceutical Sciences, Zhejiang University, Hangzhou 310058, China; 2 Collaborative Innovation Center for Diagnosis and Treatment of Infectious Diseases, First Affiliated Hospital, Zhejiang University School of Medicine, Hangzhou 310002, Zhejiang, China; 3 Zhejiang Provincial Key Laboratory of Medical Genetics, School of Life Sciences, Wenzhou Medical College, Wenzhou 325035, China; 4 Department of Engineering, School of Medicine, Tohoku University, Aoba-ku, Sendai 980-8775, Japan; 5 Laboratory of Molecular Neuropathology, Department of Pharmacology, Soochow University, College of Pharmaceutical Sciences, Suzhou 215123, China; 6 These authors contributed equally to this work

## Abstract

The role of the histamine H3 receptor (H3R) in cerebral ischaemia/reperfusion (I/R) injury remains unknown. Here we show that H3R expression is upregulated after I/R in two mouse models. H3R antagonists and H3R knockout attenuate I/R injury, which is reversed by an H3R-selective agonist. Interestingly, H1R and H2R antagonists, a histidine decarboxylase (HDC) inhibitor and HDC knockout all fail to compromise the protection by H3R blockade. H3R blockade inhibits mTOR phosphorylation and reinforces autophagy. The neuroprotection by H3R antagonism is reversed by 3-methyladenine and siRNA for *Atg7*, and is diminished in *Atg5*^*−/−*^ mouse embryonic fibroblasts. Furthermore, the peptide Tat-H3R_CT414-436_, which blocks CLIC4 binding with H3Rs, or siRNA for *CLIC4*, further increases I/R-induced autophagy and protects against I/R injury. Therefore, H3R promotes I/R injury while its antagonism protects against ischaemic injury via histamine-independent mechanisms that involve suppressing H3R/CLIC4 binding-activated autophagy, suggesting that H3R inhibition is a therapeutic target for cerebral ischaemia.

## Abstract (web summary)

Histamine H3 receptor dysregulation is a hallmark of pathological conditions in the central nervous system, and H3 receptor antagonism is neuroprotective. Here Chen *et al.* show that histamine-independent H3 receptor activation can enhance neuronal cell death during cerebral ischaemia by suppressing autophagy.

## Graphical abstract

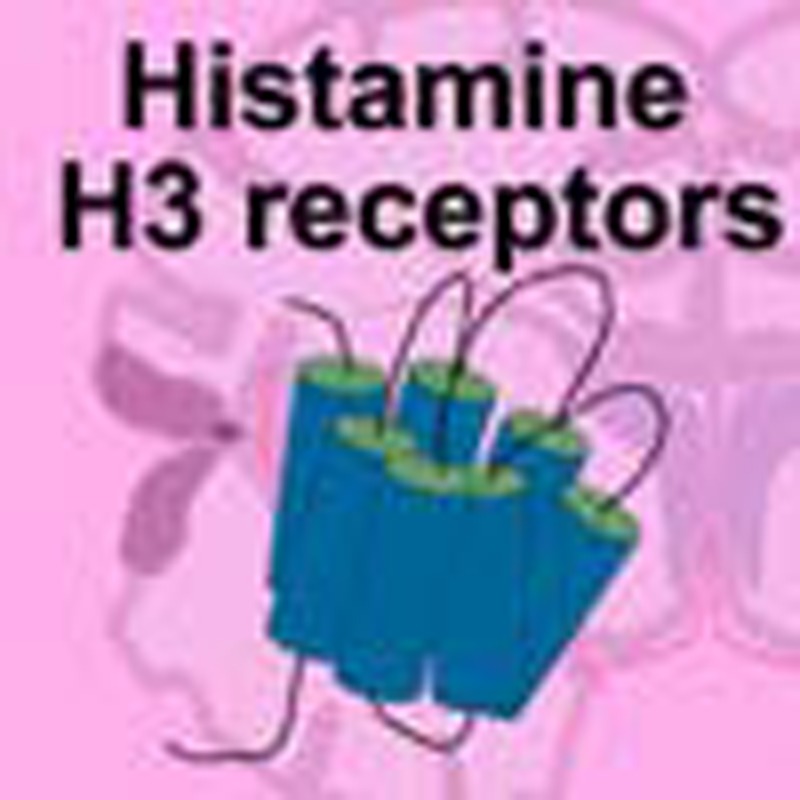

Ischaemic stroke is one of the leading causes of death worldwide^1^. Few therapies are effective other than tissue plasminogen activator, which promotes reperfusion and improves the long-term clinical outcome^2^ with limitations^3^. Despite the fact that many neuroprotective agents, including AMPA antagonists, *N*-methyl-D-aspartate antagonists and 5-hydroxytryptamine_1A_ agonists, have been developed in attempts to cure ischaemic stroke, the results have been unimpressive for a variety of reasons. Therefore, more effort is needed to elucidate the mechanisms underlying ischaemia and further explore new neuroprotective strategies.

Histamine is an endogenous neurotransmitter in the brain^4^. To date, four subtypes of receptors have been identified: H1R, H2R, H3R and H4R, of which H1R–H3R are found in brain^5^,^6^,^7^. H3R (histamine H3 receptor) is a presynaptic autoreceptor that regulates histamine release from histaminergic neurons via negative feedback^8^,^9^, as well as a heteroreceptor on non-histaminergic neurons that regulates the release of many other neurotransmitters^10^,^11^,^12^,^13^. H3R is a G-protein-coupled receptor that activates G_i/o_ proteins to inhibit adenylyl cyclase activity and modulate phospholipase A2 and mitogen-activated protein kinase activity^14^. In primary cultured rat cortical neurons, H3R activates the Akt/glycogen synthase kinase 3β (GSK-3β) axis both in a constitutive and an agonist-dependent manner^15^. Dysregulation of the downstream signalling of H3R in the Akt/GSK-3β pathway is linked to several prevalent pathological conditions^16^. H3R antagonism blocks neuropathic pain in rats^17^,^18^, electrically induced convulsions^19^ and amygdaloid-kindled seizures^20^. In addition, our previous data showed that H3R antagonism protects against *N*-methyl-D-aspartate-induced neurotoxicity in cultured cortical neurons^21^. H3R antagonism also inhibits ischaemia-induced oxidative stress^22^. Therefore, it has been proposed that H3R blockade is generally neuroprotective. However, few studies have investigated its role in ischaemia and the underlying mechanisms.

After ischaemia/reperfusion (I/R) injury, most cells in the penumbra undergo apoptosis^23^. Recently, investigations showed that autophagy also participates in the pathological process of ischaemia^24^,^25^. Autophagy is a major cellular process for the degradation of long-lived proteins and cytoplasmic organelles in eukaryotic cells^26^. Studies show that inhibition of GSK-3β, a downstream kinase of H3R, stimulates mammalian target of rapamycin (mTOR) signalling and thus inhibits autophagy^27^. Although the role of autophagy in ischaemia remains controversial, recent reports indicate that it is neuroprotective in moderate injury^24^,^26^,^27^,^28^, and these investigations have given rise to the proposal that the injury can be rescued by inducing autophagy^28^.

Therefore, the present study is designed to investigate the role of H3R in I/R injury *in vivo* and *in vitro*. We find that H3R antagonism and *H3R* deletion protect against I/R injury in a histamine-independent manner. H3R antagonists reinforces I/R-induced autophagy and the neuroprotective effect of H3R antagonism is reversed by autophagy blockade. Further investigations show that H3R antagonism decreases the binding of H3R to chloride intracellular channel 4 (CLIC4), and thus further increases autophagy. Therefore, H3R aggravates ischaemic brain injury by histamine-independent mechanisms, and its antagonism may provide a novel neuroprotective strategy against cerebral ischaemia by regulating autophagy.

## Results

### H3R antagonism protects against I/R-induced neuronal injury

**Figure 1:**
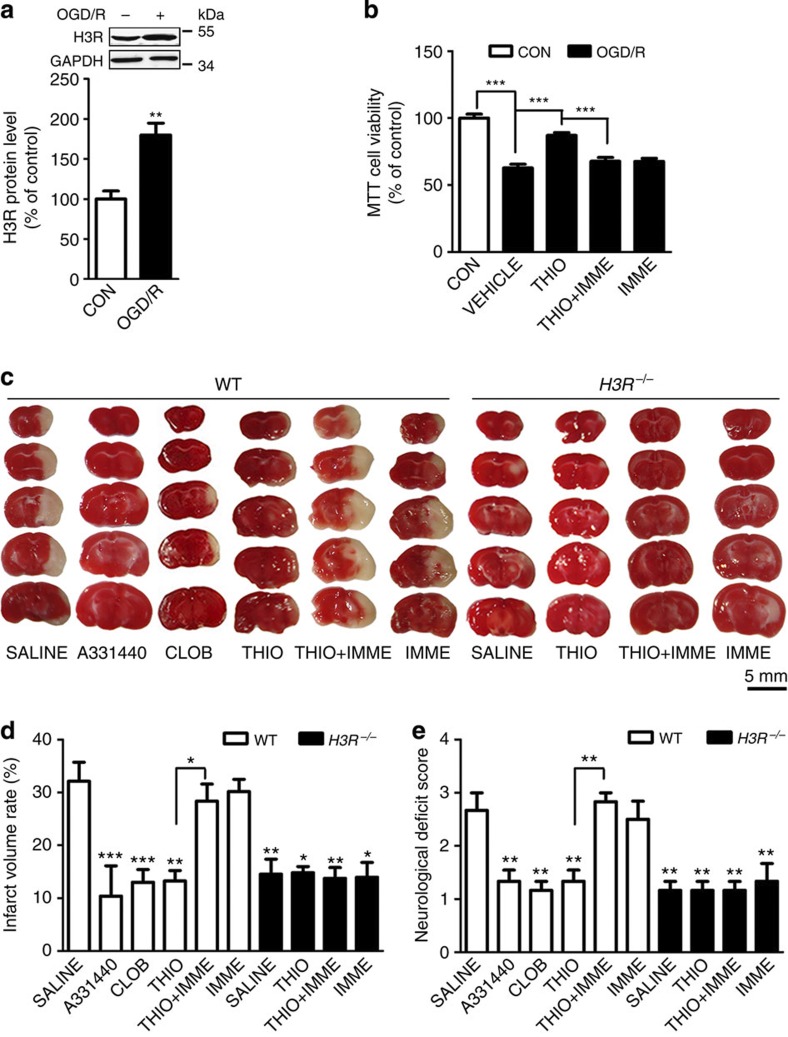
Inhibition of H3R protects against cerebral I/R-induced neuronal injury. (**a**) H3R expression in cultured rat cortical neurons at DIV7 after OGD/R injury (2 h OGD and 24 h reperfusion) shown by western blot (*n*=6 per condition; **P<0.01 versus control with Student’s *t-*test). Full-size blots are shown in Supplementary Fig. 9. (**b**) When the H3R antagonist thioperamide (THIO, 10^−6^ mol l^−1^ at reperfusion) and the agonist immepip (IMME, 10^−6^ mol l^−1^ at reperfusion) were administered, viability was tested in cultured neurons by 3-(4,5-dimethylthiazol-2-yl)-2,5-diphenyltetrazolium bromide (MTT) assay after OGD/R (*n*=7 per condition; ***P<0.001 with analysis of variances (ANOVAs) followed by the Bonferroni/Dunn *post hoc* test). (**c**) 2,3,5-triphenyltetrazolium chloride-stained brain sections showing the infarct area in WT and *H3R*^*−/−*^ mice receiving saline, the H3R antagonists A331440, clobenpropit (CLOB) and thioperamide, (THIO; 5 mg kg^−1^, i.p., immediately at reperfusion and 6 h later), and the H3R agonist immepip (IMME, 1 μg, i.c.v., at reperfusion) after I/R (1 h MCAO followed by 24 h reperfusion; scale bar, 5 mm). The bar graphs show infarct volume (**d**) and neurological score (**e**) (*n*=6–8 mice per condition, *P<0.05, **P<0.01, ***P<0.001 versus WT saline group with ANOVAs followed by the Bonferroni/Dunn *post hoc* test). Data are presented as mean±s.e.m.

Cultured rat cortical neurons exposed to oxygen-glucose deprivation/reperfusion (OGD/R) and mice with transient middle cerebral artery occlusion (tMCAO) were used. In the cultured neurons, the H3R expression increased markedly after OGD/R (2 h OGD followed by 24 h reperfusion; Fig. 1a). In H3R-transfected HEK293 cells, serum deprivation increased cell death versus the control vector group (Supplementary Fig. 1). In neurons after OGD/R, the viability declined to 62.84±2.70% of control (Fig. 1b, and thioperamide (H3R antagonist, 10^−6^ mol l^−1^) rescued the viability to 87.07±2.14% (*n*=7, *P*<0.001), which was reversed by immepip (H3R agonist, 10^−6^ mol l^−1^) to 67.82±2.75% (*n*=7 *P*<0.001, Fig. 1b). All results are expressed as mean±s.e.m. Statistical analysis was performed by two-tailed Student’s *t*-test or analysis of variance, followed by the Bonferroni/Dunn *post hoc* comparisons.

To investigate the role of H3Rs in cerebral ischaemia, they were blocked after tMCAO. The neurological deficit scores increased in wild-type (WT) mice after 24 h of reperfusion. H3Rs were blocked by the imidazole-containing antagonists, thioperamide and clobenpropit, or the non-imidazole antagonist A331440 (intraperitoneal (i.p.), 5 mg kg^−1^ administered twice; immediately at reperfusion and 6 h later). Thioperamide ameliorated the neurological deficiency, and the protection was reversed by the H3R agonist immepip. Clobenpropit and A331440 gave similar results (Fig. 1e). In support, I/R-injured *H3R*^*−/−*^ mice showed alleviated neurological deficiency (*P*<0.01, Fig. 1e), yet this was not reversed by either thioperamide or immepip. The tMCAO-induced infarct volume was 32.13±3.60% in saline-treated WT mice. The H3R antagonists thioperamide, clobenpropit and A331440 reduced the volume to 13.24±1.98, 13.00±2.43 and 10.39±5.74%, respectively (Fig. 1c,d). The reduced infarct volume was reversed by immepip to 28.34±3.25%. Moreover, in *H3R*^*−/−*^ mice, in which the basic cerebral blood flow (CBF) was unchanged, I/R induced a smaller infarct volume (reduced to 14.55±2.83% versus WT mice; *P*<0.01; *n*=6–8; Fig. 1c,d) and the protection was not affected by either H3R antagonists or agonists.

### H3R inhibition reduces I/R-induced apoptosis

**Figure 2:**
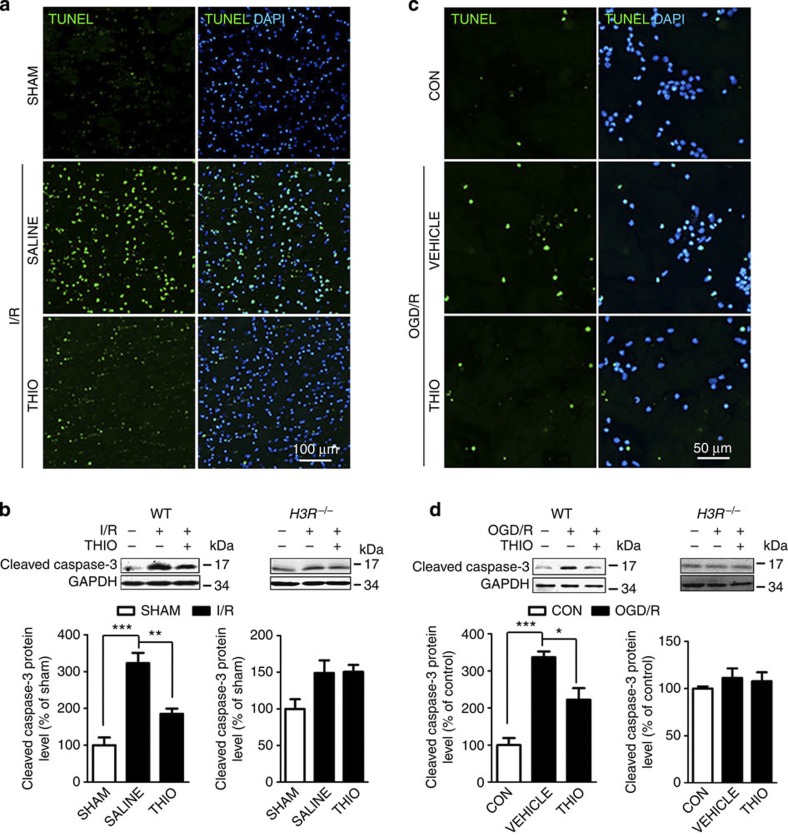
Inhibition of H3R ameliorates apoptosis induced by I/R *in vivo* and OGD/R *in vitro*. (**a**) TUNEL staining of apoptotic cells in penumbral brain slices *in vivo* (scale bar, 100 μm) and (**c**) in cultured neurons when H3R was inhibited with thioperamide (scale bar, 50 μm) after tMCAO and OGD/R. TUNEL-positive cells are green, and all cells are stained with 4',6-diamidino-2-phenylindole (blue). (**b**,**d**) H3R was inhibited by thioperamide, and cleaved caspase-3 protein expression is shown by western blot in WT and H3R^*−/−*^mice after tMCAO (**b**) (*n*=6–7 per condition; **P<0.01, ***P<0.001 with analysis of variances (ANOVAs) followed by Bonferroni/Dunn *post hoc* test) and in cultured neurons subjected to OGD/R (**d**) (*n*=8 per condition; *P<0.05, ***P<0.001 with ANOVAs followed by the Bonferroni/Dunn *post hoc* test). Data are presented as mean±s.e.m. Full-size blots are shown in Supplementary Fig. 10.

Apoptosis was determined by terminal deoxynucleotidyl transferase dUTP nick-end labelling (TUNEL) staining and cleaved caspase-3 protein levels in the cortical penumbra 24 h after I/R. The percentage of TUNEL-positive cells increased from 1.54±0.41 to 40.54±3.79% in I/R, and the ratio returned to 15.94±2.07% with thioperamide treatment (Fig. 2a). Western blot analysis showed a marked increase of cleaved caspase-3, while thioperamide reduced its expression (*P*<0.01, Fig. 2b). However, in *H3R*^*−/−*^ mice, thioperamide failed to reverse the I/R-induced caspase-3 upregulation (Fig. 2b). Similarly, OGD/R increased the TUNEL-positive neuron ratio, which was reversed by thioperamide (Fig. 2c). The cleaved caspase-3 expression also increased (to 336.70±15.71% of control) *in vitro*, while thioperamide significantly reduced the value to 222.48±30.99%. However, in cultured *H3R*^*−/−*^ neurons, OGD/R did not induce apoptosis in the presence or absence of thioperamide (Fig. 2d).

### Neuroprotection by H3R antagonists is histamine independent

**Figure 3:**
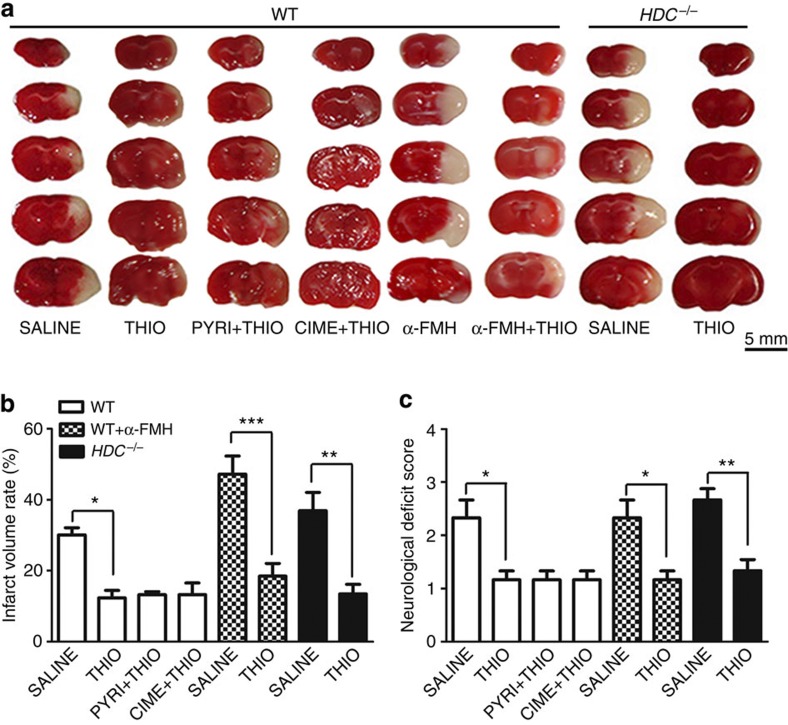
Protection by H3R inhibition is not dependent on histamine. (**a**) 2,3,5-Triphenyltetrazolium chloride (TTC)-stained brain sections from WT and *HDC*^*−/−*^ mice showing the infarct area in those receiving saline, the H3R antagonist thioperamide (THIO), the H1R antagonist pyrilamine (PYRI; 10 mg kg^−1^, i.p., at reperfusion), the H2R antagonist cimetidine (CIME; 10 mg kg^−1^, i.p., at reperfusion) and α-FMH (50 mg kg^−1^, i.p., 2 h before ischemia) after 24 h reperfusion (*n*=6 per condition; scale bar, 5 mm). (**b**) Infarct volumes and (**c**) neurological deficit scores (*P<0.05, **P<0.01, ***P<0.001 with analysis of variances followed by the Bonferroni/Dunn *post hoc* test). Data are presented as mean±s.e.m.

As a presynaptic receptor on histaminergic neurons, H3R suppresses histamine synthesis and releases in a negative feedback manner^8^. Thus, inhibition of H3R by thioperamide leads to synaptic histamine release^29^. Therefore, we asked whether the protection conferred by H3R inhibition is histamine dependent, and found that the neuroprotection by thioperamide was not reversed by pyrilamine (10 mg kg^−1^) or cimetidine (10 mg kg^−1^), H1R and H2R antagonists in WT mice (Fig. 3a,b). Moreover, α-fluoromethylhistidine (α-FMH; 50 mg kg^−1^ i.p., administered 2 h before ischaemia), a selective histamine synthesis inhibitor by irreversibly inhibiting histidine decarboxylase (HDC), did not reverse the neuroprotection of thioperamide as shown by the neurological deficit score (Fig. 3c), as well as the infarct volume in WT mice (Fig. 3a,b). α-FMH is reported to maximally decrease histamine activity 2 h after administration and this lasts for 4 days in mice^30^. More importantly, in *HDC*^*−/−*^ mice, the protection by thioperamide remained, as revealed by the reduced neurological score and infarct volume (*P*<0.01; Fig. 3).

### H3R antagonism reinforces I/R-induced autophagy

**Figure 4:**
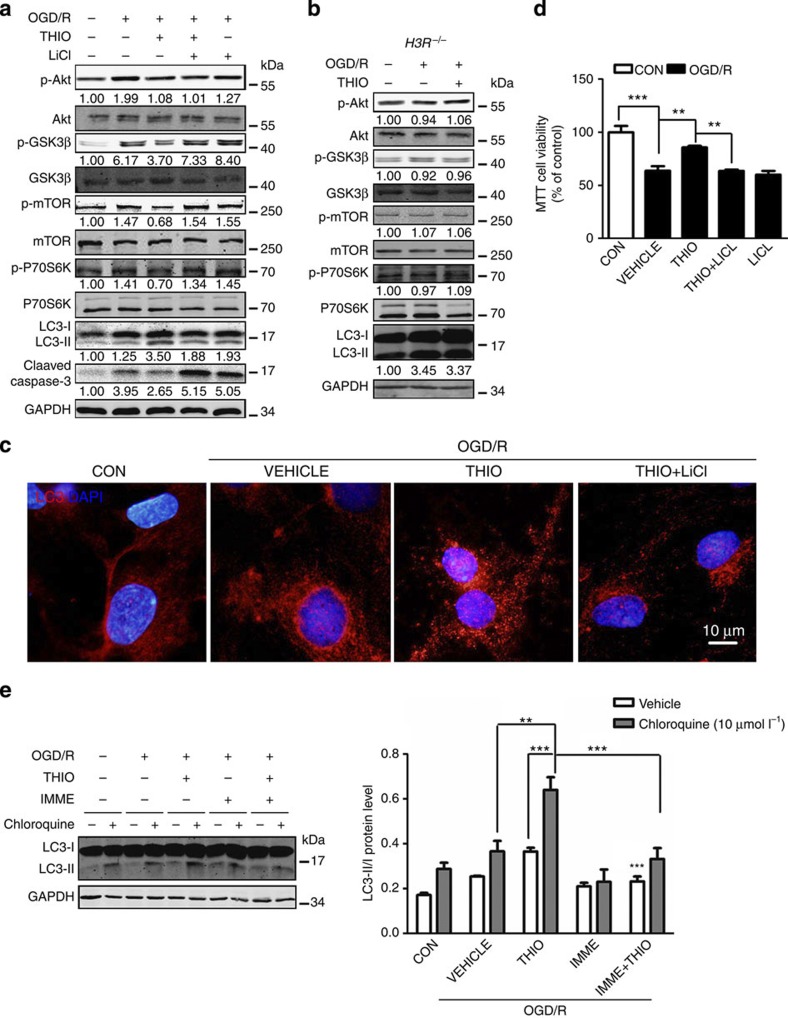
H3R inhibition activates autophagy by decreasing phosphorylation of the Akt/GSK-3β/mTOR/P70S6K signaling pathway during OGD/R. (**a**) Akt/GSK-3β/mTOR/P70S6K phosphorylation assessed by western blots after thioperamide administration and OGD/R. LiCl (5 mmol l^−1^ immediately at reperfusion) was used to rephosphorylate GSK-3β/mTOR/P70S6K. The digits below represent the semiquantified optidensity of the bands, the control bands were defined as 1.00. (**b**) Representative western blots showing the phosphorylation of Akt/GSK-3β/mTOR/P70S6K signaling in *H3R*^*−/−*^ mice after OGD/R injury. The digits below represent the semiquantified optidensity of the bands, the control bands were defined as 1.00. (**c**) Representative images showing LC3-positive autophagic vacuoles (red) with 4',6-diamidino-2-phenylindole staining (blue) after administration of thioperamide and LiCl with OGD/R (scale bar, 10 μm). (**d**) Cell viability assessed by MTT when thioperamide and LiCl were administered after OGD/R (*n*=7, **P<0.01, ***P<0.001 with analysis of variances (ANOVAs) followed by the Bonferroni/Dunn *post hoc* test). (**e**) Representative western blots and bar graph showing the expression of LC3-I and LC3-II in steady-state autophagy and autophagic flux under OGD/R injury when thioperamide (THIO) and immepip (IMME) were administered (*n*=6; **P<0.01, ***P<0.001 with ANOVAs followed by the Bonferroni/Dunn *post hoc* test). Data are presented as mean±s.e.m. Full-size blots are shown in Supplementary Fig. 11.

To further elucidate the mechanisms of the protective effects of H3R inhibition, we investigated the Akt/GSK-3β pathway downstream of H3R (ref. 15). In primary cultured neurons, OGD/R induced a marked phosphorylation of Akt/GSK-3β, which was dephosphorylated by thioperamide incubation during OGD/R, and mTOR/P70S6K phosphorylation was also inhibited (Fig. 4a). As expected, Akt/GSK-3β/mTOR/P70S6K was not phosphorylated significantly in cultured neurons from *H3R*^*−/−*^ mice (Fig. 4b). Lithium chloride (LiCl, a GSK-3β inhibitor, 5 mmol l^−1^ at reperfusion)^31^ reversed the inhibitory effect of thioperamide on Akt/GSK-3β/mTOR/P70S6K phosphorylation (Fig. 4a), and subsequently counteracted the thioperamide-rescued cell viability (Fig. 4d) and cleaved caspase-3 expression (Fig. 4a).

**Figure 5:**
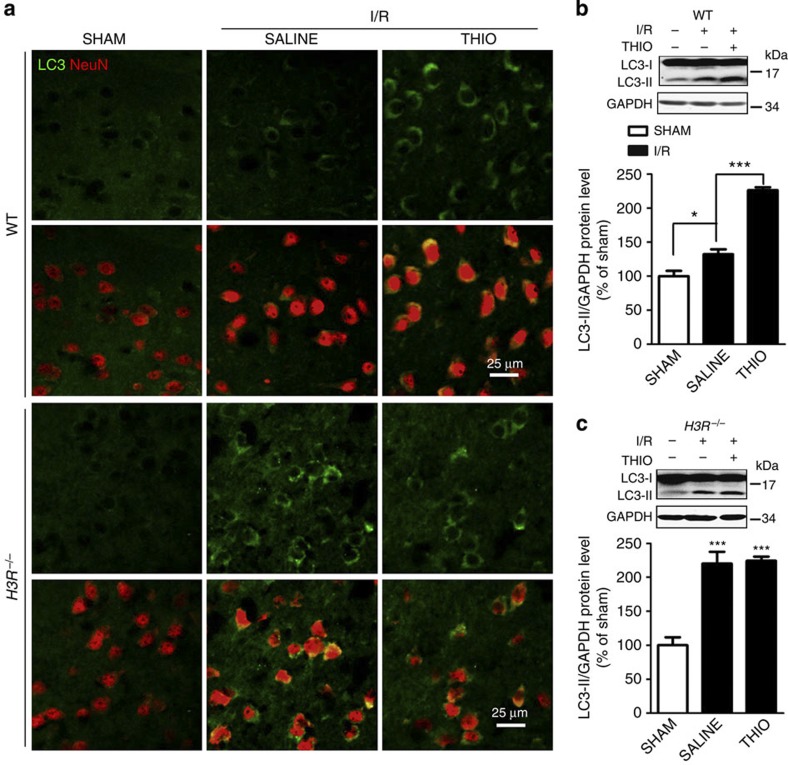
Inhibiting H3R increases autophagy *in vivo*. (**a**) Representative immunohistochemical staining of LC3 (green) along with NeuN staining (red) in WT and *H3R*^*−/−*^ mice (scale bar, 25 μm). (**b**,**c**) When thioperamide was administered *in vivo*, LC3 expression was assessed by western blot after I/R in WT mice (**b**) (*P<0.05, ***P<0.001; *n*=6 mice per condition, with analysis of variances (ANOVAs) followed by the Bonferroni/Dunn *post hoc* test), and in *H3R*^*−/−*^ mice (**c**) (***P<0.001 versus *H3R*^*−/−*^ sham group; *n*=5–6 mice per condition, with ANOVAs followed by the Bonferroni/Dunn *post hoc* test.) Data are presented as mean±s.e.m. Full-size blots are shown in Supplementary Fig. 12.

Interestingly, we found an autophagy-promoting effect of H3R inhibition, evidenced by increased LC3-II expression and autophagic vacuoles (Fig. 4a,c), both of which were reversed by LiCl. The increase of LC3-II was not due to lysosome dysfunction since autophagic flux was determined by chloroquine, a lysosome inhibitor, suggesting autophagy activation by H3R antagonism (Fig. 4e). This was further confirmed in WT mice with I/R, which showed that H3R inhibition also augmented autophagy in the penumbra as revealed by LC3-II expression (Fig. 5a,b). Moreover, we examined the effect of the H3R agonist immepip on autophagy and found that autophagy activation by thioperamide was significantly reversed by immepip. This result indicated a regulatory effect of H3R antagonism on autophagy activation. In addition, in *H3R*^*−/−*^ mice, autophagy was more robustly activated by I/R; however, further activation by thioperamide disappeared (Fig. 5a,c). Further investigations showed that the autophagy induced in I/R mainly occurred in neurons but not microglia or astrocytes (Fig. 5a; Supplementary Fig. 2).

### Autophagy suppression reverses protection by H3R antagonism

**Figure 6:**
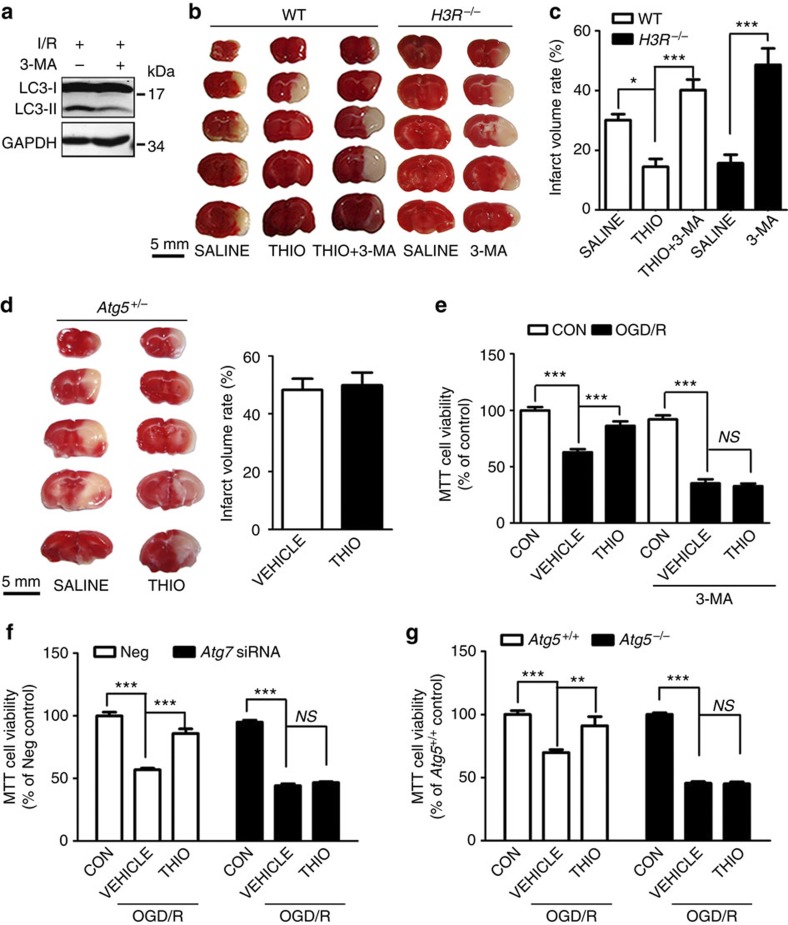
Protection by H3R inhibition is reversed by suppressing autophagy. (**a**) Western blots showing the inhibitory effect of 3-MA on autophagy in I/R. Full-size blots are shown in Supplementary Fig. 13. (**b**) TTC-stained sections showing the infarct area after administration of 3-MA (100 nmol, i.c.v., at reperfusion) in WT and *H3R*^*−/−*^ mice (scale bar, 5 mm), and (**c**) bar graph showing infarct volume (*n*=6–8 mice per condition; *P<0.05, ***P<0.001, with analysis of variances (ANOVAs) followed by the Bonferroni/Dunn *post hoc* test). (**d**) TTC-stained sections (scale bar, 5 mm) and bar graph showing the infarct area after administration of thioperamide in *Atg5*^*+/−*^mice (*n*=4–6 mice per condition). (**e**) In cultured neurons, viability was tested by MTT assay after 3-MA (2.5 mmol l^−1^ at reperfusion) and thioperamide was administered in OGD/R (*n*=7 per condition; ***P<0.001, with ANOVAs followed by the Bonferroni/Dunn *post hoc* test). (**f**) In cultured neurons, viability was tested by MTT after siRNA for *Atg7* and OGD/R (*n*=7 per condition; ***P<0.001, with ANOVAs followed by the Bonferroni/Dunn *post hoc* test). (**g**) Viability was tested by MTT assay on *Atg5*^*+/+*^and *Atg5*^*−/−*^ MEFs after thioperamide administration after OGD/R (*n*=7 per condition; **P<0.01, ***P<0.001, with ANOVAs followed by the Bonferroni/Dunn *post hoc* test). Data are presented as mean±s.e.m.

3-Methyladenine (3-MA, 100 nmol, intracerebroventricular (i.c.v.), at reperfusion, Fig. 6a) reversed the ameliorated infarct volume by H3R inhibition in WT mice (Fig. 6b,c) and in *H3R*^*−/−*^ mice (increased from 15.67±2.87 to 48.66±5.47%, *P*<0.001, Fig. 6b,c). Furthermore, thioperamide showed no significant alleviation of infarct volume in I/R injury in the *Atg5*^*+/−*^ mice (48.30±3.84% with vehicle versus 49.88±4.39% with thioperamide, *P*>0.5, Fig. 6d). We confirmed this with cultured neurons. There were no differences in cell viability between the vehicle and thioperamide groups after 3-MA administration (2.5 mmol l^−1^, at reperfusion), showing that 3-MA alone was not neurotoxic. Increased cell viability under OGD/R injury by thioperamide was observed in vehicle-treated groups but not with 3-MA treatment (Fig. 6e). Although 3-MA is a widely accepted autophagy inhibitor by blocking PI3K-class III, its non-selective effects cannot be completely excluded. To further clarify the role of autophagy in the protection by thioperamide, the key autophagy gene *Atg7* was knocked down by small-interfering RNA (siRNA). The silence effects are confirmed by western blot of LC3. (Supplementary Fig. 3). The results showed that the neuroprotection by thioperamide was diminished by *Atg7* knockdown (44.23±1.57% with vehicle versus 46.65±0.81% with thioperamide; Fig. 6f). Further, *Atg5*^*−/−*^ mouse embryonic fibroblasts (MEFs) were investigated. H3R protein and messenger RNA were detectable both in WT and *Atg5*^*−/−*^ MEFs (Supplementary Fig. 4). In WT MEFs, thioperamide protected cells against OGD/R, whereas in *Atg5*^*−/−*^ MEFs, the protection disappeared (Fig. 6g).

### CLIC4 is involved in H3R antagonism-enhanced autophagy

**Figure 7:**
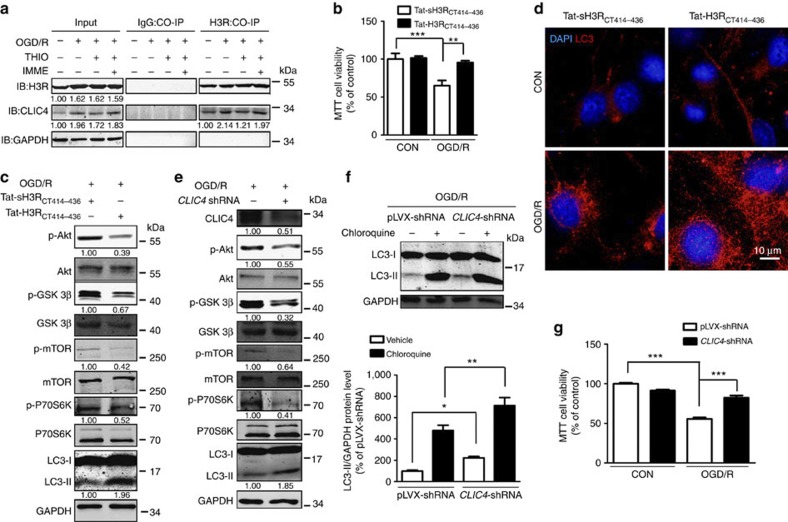
Elevated autophagy by H3R inhibition involves suppression of the interaction of H3R with CLIC4. (**a**) Representative co-immunoprecipitation results showing the effect of thioperamide on the interaction of H3R with CLIC4 during OGD/R. Full-size blots are shown in Supplementary Fig. 14. (**b**) Cell viability was tested by MTT assay after administration of Tat-H3R_CT414–436_ (10^−6^ mol l^−1^ at reperfusion) under OGD/R (data are presented as mean±s.e.m.; *n*=7 per condition; *P<0.05, **P<0.01, with analysis of variances (ANOVAs) followed by the Bonferroni/Dunn *post hoc* test). (**c**) Representative western blots showing the effect of Tat-H3R_CT414-436_ on phosphorylation of Akt/GSK-3β/mTOR/P70S6K signalling and LC3 expression in OGD/R. Full-size blots are shown in Supplementary Fig. 14. (**d**) Representative images of the effect of Tat-H3R_CT414-436_ on LC3-positive autophagic puncta (red) in 4',6-diamidino-2-phenylindole-stained (blue) neurons after OGD/R (scale bar,10 μm). (**e**) Western blots showing the effect of siRNA for *CLIC4* on phosphorylation of Akt/GSK-3β/mTOR/P70S6K signalling and LC3 expression in OGD/R. Full-size blots are shown in Supplementary Fig. 14. (**f**) Representative western blots and bar graph showing the expression of LC3-I and LC3-II on steady-state autophagy and autophagic flux under OGD/R when CLIC4 was knocked down (data are presented as mean±s.e.m.; *n*=6; *P<0.05, **P<0.01; , with ANOVAs followed by the Bonferroni/Dunn *post hoc* test). Full-size blots are shown in Supplementary Fig. 14. (**g**) Cell viability was assessed by MTT assay after siRNA for *CLIC4* under OGD/R (data are presented as mean±s.e.m.; *n*=8 per condition; ***P<0.001, with ANOVAs followed by the Bonferroni/Dunn *post hoc* test).

It has been reported that H3R may function by binding with CLIC4 (Maeda *et al.*^32^), which is thought to regulate autophagy^33^. Thus, we speculated that thioperamide regulates autophagy in the context of OGD/R in a CLIC4-related manner. To test this hypothesis, we assessed CLIC4 expression and the influence of thioperamide on the interaction between H3R and CLIC4. After OGD/R, both H3R and CLIC4 total protein were upregulated, and the interaction of H3R with CLIC4 significantly increased (Fig. 7a). Co-immunoprecipitation analysis showed that thioperamide did not change the protein expression of H3R or CLIC4, but it inhibited the interaction of H3R with CLIC4, which was reversed by the H3R agonist immepip (Fig. 7a). To further clarify the role of CLIC4 in the protection by thioperamide, the peptide Tat-H3R_CT414-436_ (10^−6^ mol l^−1^ at reperfusion) was used to block the H3R-CLIC4 binding in neurons (Supplementary Fig. 5). Tat-H3R_CT414–436_ preserved the viability under OGD/R (increased from 64.92±6.92 to 95.54±2.37% of control, *P*<0.01, Fig. 7b). Moreover, Tat-H3R_CT414–436_ led to dephosphorylation in the Akt/GSK-3β/mTOR/P70S6K signalling pathway, upregulating LC3-II expression and increasing LC3-positive autophagic vacuoles (Fig. 7c,d). In addition, siRNA for *CLIC4* increased the viability under OGD/R (from 55.70±1.59 to 82.26±2.93% of control, *P*<0.001, Fig. 7g), and increased the LC3-II expression as well as the cumulative autophagy by dephosphorylating the Akt/GSK-3β/mTOR/P70S6K signalling pathway (Fig. 7e–g).

## Discussion

The H3R is found predominantly in the brain, which implies a role in brain disorders. In the present study, we demonstrated for the first time that H3R antagonism protects against I/R injury *in vivo* and OGD/R injury *in vitro*. Both H3R antagonists and H3R-knockout decreased the ischaemic damage. We also found that H3R antagonism disturbed the binding of H3R with CLIC4, which may subsequently protect cells against OGD/R injury via Akt/GSK-3β signalling. The present findings shed light on H3R and its signalling pathways in the context of ischaemic brain injury, and suggest that H3R is a potential target in therapy for cerebral ischaemia.

At present, the role of H3Rs in cerebral ischaemia has not been fully elucidated. We showed that OGD/R significantly increased H3R expression, and H3R transfection increased the vulnerability of HEK293 cells to serum deprivation (Supplementary Fig. 1). Conversely, three H3R antagonists (thioperamide, clobenpropit and A331440) protected against I/R-induced focal brain ischaemia. The protection by thioperamide was significantly reversed by the selective H3R agonist immepip, suggesting that H3R aggravates ischaemic injury. We further used *H3R*^*−/−*^ mice which, interestingly, showed ameliorated ischaemic brain injury compared with their WT littermates, and the protective effects of H3R antagonists were absent. It is likely that H3R antagonists protect ischaemic neurons by directly blocking H3Rs. Therefore, our data for the first time showed that H3R aggravates ischaemic injury and its inhibition enhances neuronal survival.

H3R is a presynaptic autoreceptor that inhibits histamine release via negative feedback, and H3R antagonism leads to increased histamine release. H1R and H2R are postsynaptic receptors responsible for protective histaminergic signalling^34^,^35^. Thus, it has been hypothesized that the beneficial effects of H3R antagonism might involve the augmentation of central histaminergic activity^34^,^36^,^37^. To our surprise, neither the H1R antagonist pyrilamine nor the H2R antagonist cimetidine compromised the protection by thioperamide. We demonstrated that thioperamide-induced histamine release was not sufficient to rescue ischaemic brain injury even in the presence of metoprine, which inhibits histamine degradation^38^. So, it is likely that increased histamine does not underlie the protection by H3R antagonists. To provide more solid evidence, we showed that both α-FMH, a selective HDC inhibitor that decreases histamine synthesis^30^ (Supplementary Fig. 6), and *HDC* knockout failed to reverse the protection by thioperamide. In addition, it has been reported that *H3R*^*−/−*^ mice show a significantly decreased brain histamine level^39^. Nevertheless, the ameliorated I/R injury was still evident in the present data, which further supported our notion that histamine is not involved in the protection by H3R antagonism. Taken together, these results suggested that a new non-histaminergic mechanism exists in the protection against ischaemic brain injury.

Studies have revealed that brain H3Rs couple with G_i/o_ proteins by which protein kinases such as cyclic AMP-dependent protein kinase A and GSK-3β are modulated^15^,^40^. The present work found that LiCl (a GSK-3β inhibitor) completely reversed the protection by thioperamide, indicating that activation of GSK-3β may be necessary for the protection by H3R antagonism. It has been reported that I/R-induced activation of Akt, an upstream kinase of GSK-3β, may ultimately lead to neuronal injury^41^, supporting our results that Akt/GSK-3β phosphorylation was deleterious to neuronal survival. Our results are also supported by a recent finding that dephosphorylation of GSK-3β protects against prolonged myocardial ischaemic injury^27^. In addition, it was previously reported that an H3R agonist protects neurons against neuroexcitotoxicity by reversing the dephosphorylation of the Akt/GSK-3β pathway^42^, whereas we found that Akt/GSK-3β was significantly phosphorylated in OGD/R injury (Fig. 4a), which suggests that different mechanisms may be involved in the two cell-injury models. Thioperamide may be protective in targeting I/R-induced cell injury that involves phosphorylated Akt/GSK-3β.

Interestingly, the activation of GSK-3β by H3R inhibition subsequently inhibits mTORC1 activity, as revealed by decreased phosphorylation of p70S6K^43^. The mTORC1 signalling plays a key role in autophagy induction, and we found that both H3R inhibition and knockout enhanced autophagy in I/R. Additional data indicated that autophagy occurred primarily in neurons but neither in astrocytes nor in microglia. In addition, autophagy inhibition by 3-MA, *Atg7* silencing and *Atg5* knockout all reversed the protection by thioperamide against I/R injury, suggesting that autophagy reinforced by H3R inhibition underlies the neuroprotection. Our previous research also showed that the protective role of autophagy during I/R may be attributable to autophagy-related mitochondrial clearance and the inhibition of downstream apoptosis^44^. Moreover, our previous study showed that autophagy plays distinct roles in ischaemia and the reperfusion phase. In particular, we demonstrated that autophagy protects against reperfusion-induced neuronal injury^44^. Here we found that thioperamide only protected against I/R injury but not that induced by ischaemia alone. These findings reinforced the idea that protective autophagy is only activated during reperfusion. Therefore, our results revealed a novel protective mechanism of H3Rs. It is noteworthy that the H3R is also a heteroreceptor involved in the regulation of several neurotransmitters including glutamate, dopamine, 5-hydroxytryptamine and γ-aminobutyric acid^45^. These neurotransmitters are known to participate in the pathogenesis of cerebral ischaemia, and it has been suggested that H3R antagonism ameliorates excitotoxicity^21^. Besides, an anti-inflammatory effect of H3R antagonism has also been suggested in brain ischaemia^46^. Therefore, the involvement of other mechanisms cannot be excluded.

To further explore the mechanisms by which H3R blockade enhances autophagy, we investigated the involvement of histamine in the regulation of autophagy by H3R. Interestingly, histamine had no effect on mTOR phosphorylation and autophagy in OGD/R (Supplementary Fig. 7), which provided further evidence for our previous notion that H3R antagonism protects against ischaemic injury in a histamine-independent manner. It has been suggested that CLIC4 is an H3R-interacting protein, and Zhong *et al.*^33^ have recently reported that CLIC4 is involved in the regulation of autophagy in glioma cells^32^. Therefore, we proposed that the inhibition of H3R suppresses H3R binding with CLIC4 and subsequently induce autophagy. We found that CLIC4 expression was significantly upregulated and its binding with H3R was also increased in OGD/R neurons, suggesting that CLIC4 might be involved in the cell injury in response to stress^47^. More interestingly, H3R inhibition by thioperamide disturbed the H3R-CLIC4 interaction without altering their expression. We also determined the localization of H3R in mouse brain; it was shown that H3Rs predominantly locate in neurons in cortex, hippocampus and stratum (Supplementary Fig. 8). This effect of thioperamide was reversed by the H3R agonist immepip, indicating that CLIC4-H3R binding may, at least partly, inhibit the autophagy regulated by H3R antagonism. To clarify this, we used the peptide Tat-H3R_CT414–436_, which competitively blocks the CLIC4-H3R interaction by binding with CLIC4. We found that Tat-H3R_CT414–436_ conferred protection to an extent similar to H3R blockade in OGD/R (Fig. 7a). In addition, Tat-H3R_CT414–436_ inhibited Akt, activated GSK-3β, inhibited mTOR/P70S6K signalling and ultimately upregulated the autophagy level in the OGD/R period. Furthermore, siRNA for *CLIC4* also exhibited protection through autophagy induced by mTOR inhibition (Fig. 7). Therefore, these data strongly suggested that H3R blockade disturbs CLIC4-H3R binding and then activates autophagy to protect cells against I/R injury. Previous investigations indicated that suppression of CLIC4 may overactivate Beclin1 and thus activates autophagy, which implies it could be an underlying mechanism^33^. Further studies are needed to address this issue.

**Figure 8:**
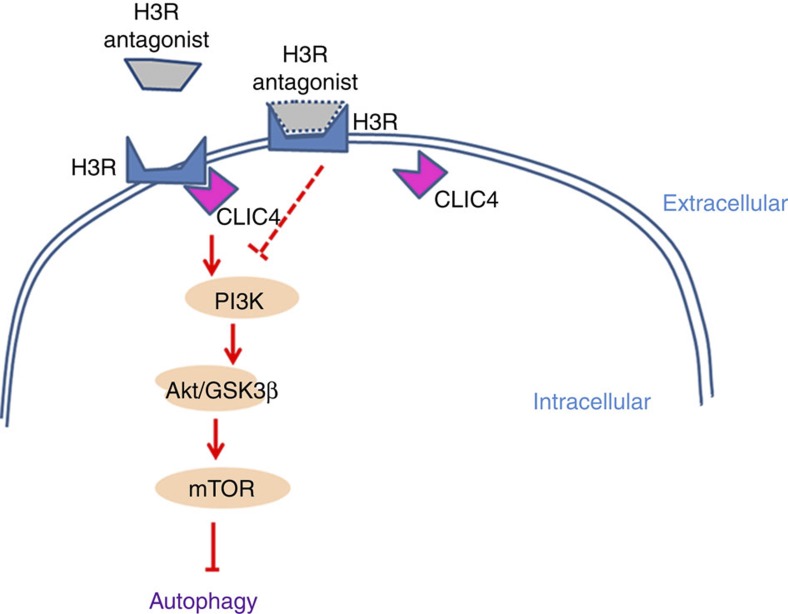
Proposed model for the neuroprotective effects of H3R antagonism in ischemic injury. H3R antagonist binds with H3R and subsequently releases its binding with CLIC4. This process inhibits PI3K/Akt/GSK-3β/mTOR signalling, that activates autophagy.

In conclusion, the present study showed for the first time that H3R enhances I/R-induced neuronal injury. H3R inhibition confers neuroprotection against ischaemia through histamine-independent mechanisms. H3R antagonism inhibits CLIC4-H3R binding and subsequently further activates I/R-induced autophagy, which protects against ischaemic injury (Fig. 8). The present study provides new prospects for H3R antagonists in therapeutic intervention for cerebral ischaemia.

## Methods

### Animals

Eight to 12-week-old WT, *HDC*^*−/−*^, *H3R*^*−/−*^ and *Atg5*^*+/−*^ male mice (all C57BL/6 strain) weighing 22–25 g were used. The *H3R*^*−/−*^ mice were supplied by Johnson and Johnson Pharmaceutical Research and Development, LLC (La Jolla, CA), bred and maintained by the Jackson Laboratory. The *HDC*^*−/−*^ mice were kindly provided by Professor Ohtsu^48^. The *Atg5*^*−/−*^ mice were obtained from the RIKEN Bioresource Center (Ibaraki, Japan). The *HDC*^*−/−*^, *H3R*^*−/−*^ and *Atg5*^*+/−*^mice were verified by reverse transcriptase–PCR. The PCR primers for the WT *HDC* gene were sense 5′-ACCCCATCTACCTCCGACAT-3′, antisense 5′-ACCGAATCACAAACCACAGC-3′ (ref. 49). The PCR primers for the WT *H3R* gene were sense 5′-CACACCCTTCCTCAGCGTTA-3′, antisense 5′-CCCTTTTGAGTGAGCGTGG-3′. The primers for *Atg5*^*+/−*^ mice were 5′-ACAACGTCGAGCACAGCTGCGCAAGG-3′, 5′-GAATATGAAGGCACACCCCTGAAATG-3′ and 5′-GTACTGCATAATGGTTTAACTCTTGC-3′. All experiments and protocols were approved by the Zhejiang University Animal Experimentation Committee and were in complete compliance with the National Institutes of Health Guide for the Care and Use of Laboratory Animals.

### Focal cerebral ischaemia

WT, *HDC*^*−/−*^, *H3R*^*−/−*^ and *Atg5*^*+/−*^ mice were fasted overnight and anaesthetized by i.p. injection of chloral hydrate (400 mg kg^−1^). Transient focal cerebral ischaemia was induced by MCAO^49^. In brief, a 6-0 nylon monofilament suture, blunted at the tip and coated with 1% poly-L-lysine, was advanced ~10 mm into the internal carotid to occlude the origin of the MCA. Reperfusion was allowed after 1 h by monofilament removal. Body temperature was maintained at 37 °C with a heat lamp (FHC, Bowdoinham, ME, USA) during surgery and for 2 h after the start of reperfusion. CBF was determined in the territory of the MCA by laser Doppler flowmetry (Moor Instruments Ltd.). A flexible fibre-optic probe was affixed to the skull over the cortex supplied by the proximal part of the right MCA (2 mm caudal to the bregma and 6 mm lateral to the midline). Animals with <80% reduction in CBF in the core of the MCA territory were excluded from the study. Neurologic deficit scores were evaluated at 24 h of reperfusion as follows: 0, no deficit; 1, flexion of the contralateral forelimb on lifting of the whole animal by the tail; 2, circling to the contralateral side; 3, falling to the contralateral side; and 4, no spontaneous motor activity^49^,^50^.

### Infarct volume measurement

Infarct volume was determined 24 h after reperfusion. The brains were quickly removed, sectioned coronally at 2-mm intervals and stained by immersion in the vital dye 2,3,5-triphenyltetrazolium hydrochloride (0.25%) at 37 °C for 30 min. The extents of the normal and infarcted areas were analysed using ImageJ (National Institutes of Health, Bethesda, MD, USA) and determined by the indirect method, which corrects for oedema (contralateral hemisphere volume minus non-ischaemic ipsilateral hemisphere volume). The percentage of the corrected infarct volume was calculated by dividing the infarct volume by the total contralateral hemispheric volume, and this ratio was then multiplied by 100.

### Cell culture and transfection

For primary neuronal cell culture, pregnant Sprague–Dawley rats or C57BL6J mice were anaesthetized by i.p. injection of chloral hydrate (400 mg kg^−1^), and the cortex was isolated from embryos (16 days) for primary cortical neuron cultures. Cells (800–1,000 cells mm^2^) were seeded on coverslips coated with 30 mg ml^−1^ poly-D-lysine. Cells were placed in fresh serum-free Neurobasal medium (21103, Gibco) plus 2% B27 and fed every 4 days with fresh medium and used after 7 days (DIV7). In addition, HEK293 cells (from the ATCC) and MEFs (kind gifts from Professor Mizushima) were cultured in DMEM with 10% fetal bovine serum (Gibco). The full-length coding domain sequence of mouse H3R was constructed into plasmid pcDNA3.1 and transfected into HEK293 cells by Lipofectamine 2,000 reagent (Invitrogen) 24 h before experiments.

### Oxygen-glucose deprivation

OGD was induced in DMEM without glucose and saturated with 5% CO_2_/95% N_2_ for 2 h for neurons, 9 h for MEFs. After OGD, 24 h reperfusion with high-glucose DMEM (4.5 g l^−1^) in normoxia was carried out. For serum starvation, HEK293 cells transfected with mouse *H3R* complementary DNA or vehicle plasmids were exposed to DMEM without serum for 24 h.

### Reagent administration

All the reagents used in the present study were from Sigma-Aldrich unless otherwise mentioned. For *in vivo* experiments, thioperamide, clobenpropit and A331440 were administered (i.p.,5 mg kg^−1^) twice; immediately at reperfusion and 6 h later. α-FMH (a kind gift from Dr Kamei Chiaki; i.p., 50 mg kg^−1^) was given 2 h before ischaemia. Immepip (i.c.v., 2 μl of 0.5 μg μl^−1^), pyrilamine or cimetidine (i.p., 10 mg kg^−1^) and 3-MA were administered (i.c.v., 2 μl of 7.5 μg μl^−1^) at reperfusion. For *in vitro* experiments, all reagents at the indicated concentrations, 3-MA (2.5 mmol l^−1^) and LiCl (5 mmol l^−1^) were added at the beginning of reperfusion after OGD. Tat-H3R_CT_ (Tat-LCHYSFRRAFTKLLCPQKLKVQP) or Tat-sH3R_CT_ (Tat-AVQKCRLPQLPLCYRHSFTKLKF; GL Biochem, Shanghai) at 1 μmol l^−1^ was also applied directly to cells at reperfusion.

### Apoptosis determined by TUNEL assay

Apoptotic cells were determined by TUNEL assay (Roche) and the total cell number was counted after DAPI (4',6-diamidino-2-phenylindole) staining. For mouse brain samples, coronal cryosections from at least five animals in each group were obtained. Five random fields in the penumbral areas of each section were observed. Cultured cells had been seeded on poly-L-lysine-treated coverslips, and at least three coverslips from each group were included in one experiment. Five random fields were observed on each coverslip, and the experiments were repeated independently three times. The results were expressed as the percentage of TUNEL+/DAPI+ cells in the sections.

### Co-immunoprecipitation and western blot

Mice were anaesthetized by i.p. injection of chloral hydrate (400 mg kg^−1^), killed 24 h after tMCAO or sham operation, the brain was quickly removed and was immediately put in −40 °C for 5 min. To dissect out the ischaemic penumbra from the ipsilateral hemisphere, the frozen brains were put on a block of pre-chilled metal. A longitudinal cut from top to bottom was made ~1 mm from the midline through the ipsilateral hemisphere. Then, a transverse diagonal cut at approximately the ‘2 o’clock’ position was made to exclude the ischaemic core region^51^. The separated tissue was lysed in ice-cold lysis buffer containing (in mmol l^−1^): 50 Tris–HCl, 150 NaCl, 1% NP-40, 2 EDTA, 1 Na_3_VO_4_, pH 7.4). Cultured neurons, MEFs and HEK 293 cells were lysed after 24 h of reperfusion. After clearing debris by centrifugation at 14,000 *g* at 4 °C, the protein concentration in the extracts was determined by the Bradford assay (Thermo, Hercules, CA). For co-immunoprecipitation, the extracts (500 μg protein) were incubated with nonspecific IgG (2 μg; ab37355, Abcam) or monoclonal antibody against H3R (2 μg; ab84468, Abcam) overnight at 4 °C, followed by the addition of 40 μl anti-rabbit protein G-agarose (ab7022, Abcam) for 3 h at 4 °C. The precipitates were washed four times with lysis buffer and denatured with SDS sample-loading buffer and separated on 12% SDS—polyacrylamide gel electrophoresis. Proteins were transferred onto nitrocellulose membranes using a Bio-Rad mini-protein-III wet transfer unit overnight at 4 °C. Transfer membranes were then incubated with blocking solution (5% non-fat dried milk dissolved in TBST buffer (in mM): 10 Tris–HCl, 150 NaCl and 0.1% Tween-20) for 1 h at room temperature, washed three times and incubated with primary antibody against CLIC4 (1:1,000, HPA008019, Sigma), H3R (1:1,000; ab84468, Abcam), p-Akt (1:1,000; #9271, CST), Akt (#9272, 1:1,000; CST), p-GSK-3β (1:1,000; #9323, CST), GSK-3β (1:1,000; #9315, CST), p-mTOR (1:1,000; #2976, CST), mTOR (1:1,000; #2983, CST), p-P70S6K (1:1,000; #9208, CST), P70S6K (1:1,000; #2708, CST), cleaved caspase-3 (1:1,000; #9661, CST), LC3 (1:1,000; PM036, MBL) and GAPDH (1:3,000; KC-5G4, KangChen Bio-tech, Shanghai) for 2 h at room temperature. Membranes were washed three times in TBST buffer and incubated with the appropriate secondary antibodies (Odyssey, LI-COR, 1:5,000 dilution) for 2 h. Images were acquired with the Odyssey infrared imaging system and analysed as specified in the Odyssey software manual. The results were expressed as the target protein/GAPDH ratio and then normalized to the values measured in the sham or control groups *in vivo* and *in vitro* (presented as 100%). The lanes marked ‘input’ were loaded with 10% of the starting material used for immunoprecipitation.

### Immunohistochemistry

Immunostaining was also performed in cultured neurons and frozen brain sections^52^,^53^. Neurons seeded on coverslips were fixed in cold methanol for 10 min and frozen brain sections were fixed in 4% paraformaldehyde for 2 h, and then incubated in 5% BSA for 2 h to block nonspecific binding of IgG. Then the cells were reacted with mouse monoclonal antibody against LC3 (1:100; M152-3, MBL), rabbit polyclonal antibody against LC3 (1:100; PM036, MBL) and mouse monoclonal antibody against NeuN (1:300; MAB377, Millipore) at 4 °C overnight. After repeated washes in PBS buffer, cells were incubated with secondary antibody in 3% BSA for 2 h at 25 °C. The secondary antibodies used in this experiment were goat anti-rabbit IgG-AlexaFluo 488 (1:300, A-11008, Invitrogen), goat anti-mouse IgG-AlexaFluo 488 (1:300, A-11001, Invitrogen) and goat anti-mouse IgG-AlexaFluo 594 (1:300, A11005, Invitrogen). After further washing in PBS, cultures were dried, cover slipped and mounted on glass slides. The stained cells were observed under a fluorescence microscope (Olympus BX51, Japan).

### RNA interference

siRNA targeting rat *Atg7* (5′-GCAUCAUCUUUGAAGUGAA-3′) and non-targeting control siRNA (5′-AUGAACGTGAAUUGCUCAA-3′) were synthesized (GenePharm, Shanghai). Primary neurons were transfected on DIV5, with 20 nmol *Atg7* or scrambled siRNA using Lipofectamine RNAiMAX (Invitrogen). After transfection in antibiotic-free medium for 8 h, cells were refreshed with normal medium. For CLIC4 silencing, the pLVX-shRNA1 plasmid targeting the 5′-TCTGGAGGTACCTGACAAA-3′ sequence of mouse *CLIC4* messenger RNA was transfected into HEK293T cells to package recombinant lentiviruses (shCLIC4-LVs, Clontech). Primary neurons were transfected with shCLIC4-LVs on DIV5 for 12 h. The efficacy of *Atg7* and *CLIC4* knockdown was assessed by western blot. Experiments were performed 72 h after transfection.

### Histamine content measurement by HPLC

Samples were de-proteinized with 0.4 mol l^−1^ perchloric acid and centrifuged at 15,000 *g* for 20 min at 4 °C. Then, the supernatant was removed and filtered through a 0.22-μm polyvinylidene difluoride membrane. Analysis of histamine in the supernatant was performed by HPLC (ESA, Chelmsford, MA, USA). The system consists of model 582 pump and four channel CoulArray electrochemical detector. After reacting with the derivate *o*-phthalaldehyde, analytes were separated on a 3-μm, 3 × 50 mm^2^ Capcell Pak MG C18 column (Shiseido, Japan). A two-component gradient elution system was used, with component A of the mobile phase being 100 mmol l^−1^ Na_2_HPO_4_, 13% acetonitrile and 22% methanol, pH 6.8, and component B being similar to A except with 5.6% acetonitrile and 9.4% methanol. A gradient elution profile was used as follows: 0–3.5 min, isocratic 100% B; 3.5–20 min, linear ramp to 0% B; 20–22 min, isocratic 0% B; 22–23 min, linear ramp to 100% B; 23–30 min, isocratic 100% B. The flow rate was set to 0.75 ml min^−1^. The temperature of the column was maintained at 38 °C. The data were acquired and analysed using CoulArray software.

### Statistical analyses

Results are expressed as mean±s.e.m. Statistical analysis was performed by two-tailed Student’s *t-*test or analysis of variances, followed by the Bonferroni/Dunn *post hoc* comparisons, using Prism software. A two-tailed *P* value<0.05 was considered statistically significant.

## Author contributions

H.Y., X.Z. and Z.C. participated in the design, experiments, interpretation of the results and manuscript writing; they also participated in the data analysis. Z.C., H.O., G.W., W.Hu., Y.S. and J.L. revised the manuscript and provided technical and supervisory support. W. Hou carried out animal care.

## Additional information

**How to cite this article:** Yan, H. *et al.* Histamine H3 receptors aggravate cerebral ischaemic injury by histamine-independent mechanisms. *Nat. Commun.* 5:3334 doi: 10.1038/ncomms4334 (2014).

## Supplementary Material

Supplementary Information Supplementary Figures 1-18
