# Supplementary material for: Histamine H3 receptors aggravate cerebral ischaemic injury by histamine-independent mechanisms
Source: Nat Commun. 2014 Feb 25;5:3334. doi: 10.1038/ncomms4334 (PMC3948077; doi:10.1038/ncomms4334)
Supplement: Supplementary Information — Supplementary Figures 1-18 [file ncomms4334-s1.pdf]

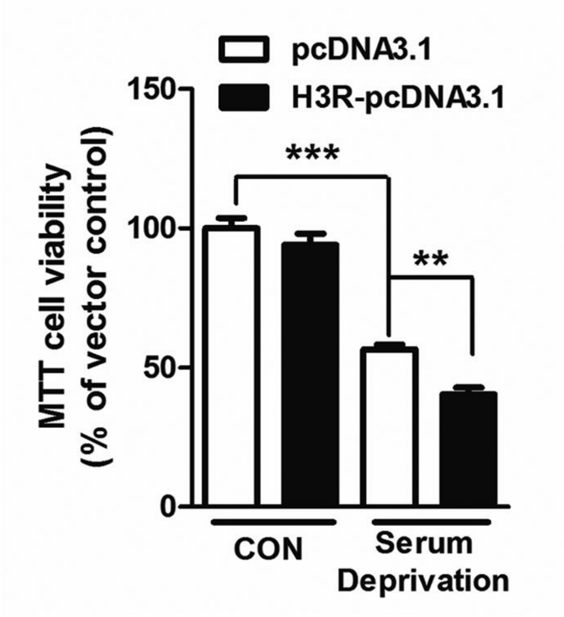

Supplementary Fig. 1 Transfection of H3R aggravates the cell injury. HEK293 cells were either transfected with H3R or empty vector. After 24 h serum deprivation, cell viability was assessed by MTT. (n = 7 ****P*<0.001; ***P* <0.01 *vs*. indicated groups, with ANOVAs followed by Bonferroni/Dunn *post-hoc* test). Data are presented as mean ± SEM.

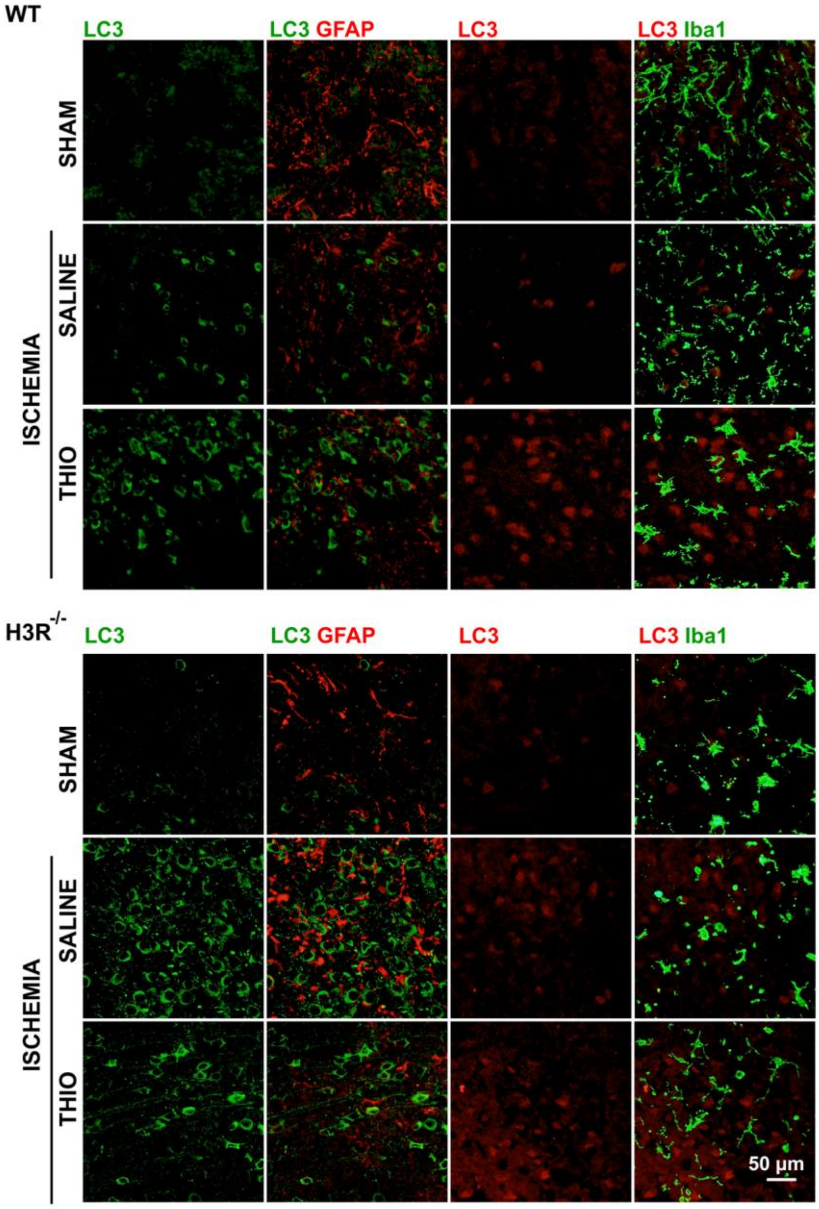

Supplementary Fig. 2 Autophagy is not examined in astrocytes and microglia in ischemic brains. Representative images showed LC3 (green) and double staining with either astrocyte marker GFAP (red) or microglial marker Iba-1 (red) in WT (upper panel) and *H3R*$^{-/-}$ (lower panel) mice with indicated treatment. (scale bar, 50 μm).

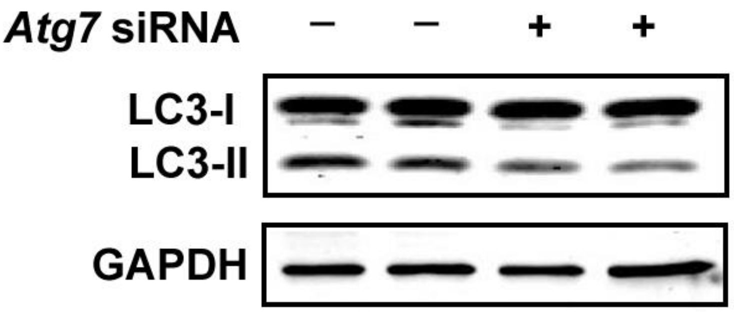

Supplementary Fig. 3 The Atg7 silence effects are confirmed by western blot detecting of LC3.

Primary cultured neurons were transfected on DIV5, with 20 nmol *Atg7* (+) or scrambled siRNA (-) using Lipofectamine RNAiMAX (Invitrogen). Full-size blots are shown in Supplementary Fig. 15.

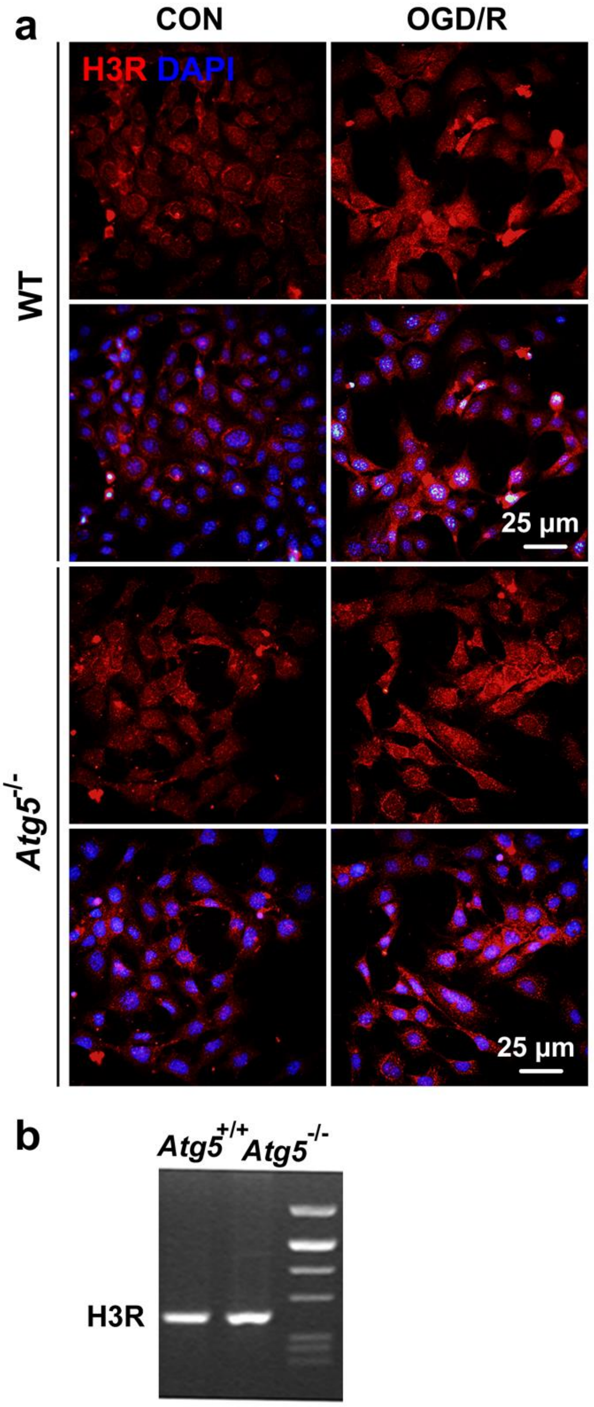

Supplementary Fig. 4 ***H3R*** **mRNA expression in MEFs.** (**a**) Representative images of H3R immunostaining in WT and *Atg5*$^{-/-}$ MEFs after 9 h OGD and 24 h reperfusion. Cells were double-stained with DAPI (blue, lower panels) (scale bar, 50 μm). (**b**) RT-PCR showed the *H3R* mRNA was expressed in MEFs. Full-size gel is shown in Supplementary Fig. 16

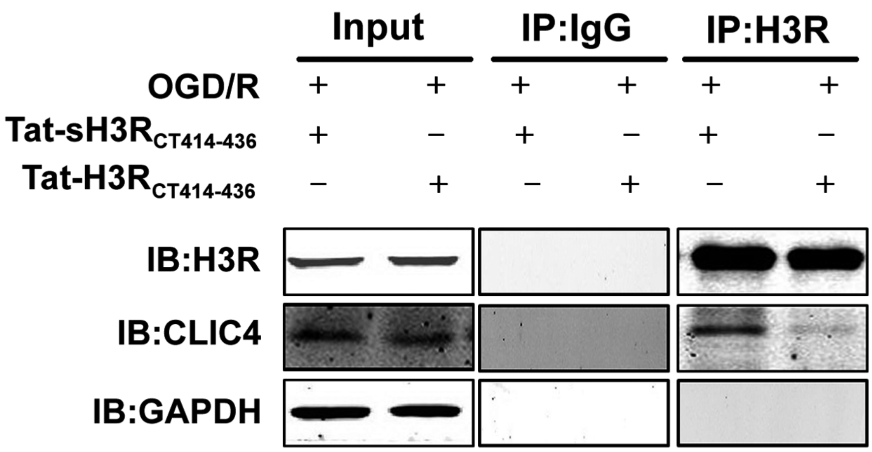

Supplementary Fig. 5 Tat-H3R$_{CT414-436}$ disturbs the interaction between H3R and CLIC4.

Co-immunoprecipitation experiment showing that Tat-H3R$_{CT414-436}$ inhibited the interaction of H3R with CLIC4. Full-size blots are shown in Supplementary Fig. 17.

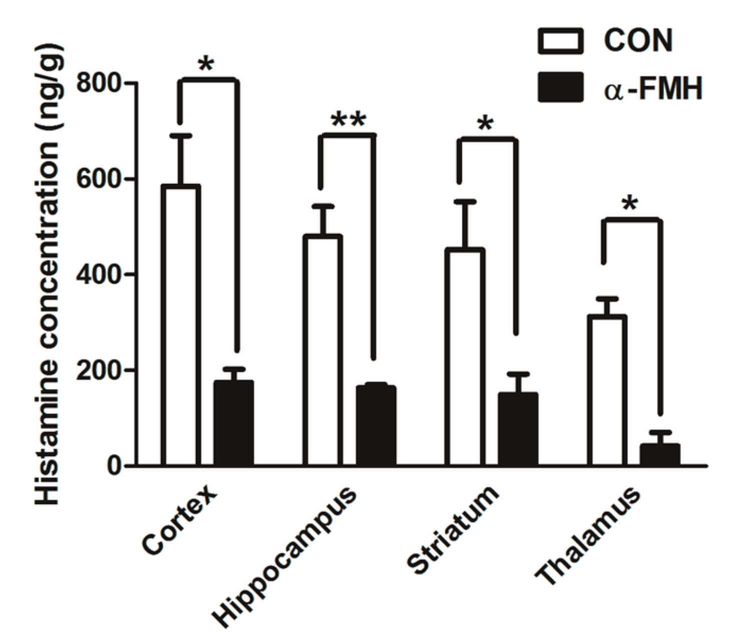

Supplementary Fig. 6 α-FMH decreases the histamine level in different brain regions. As it was shown by HPLC, the histamine level reduced significantly in every brain regions when α-FMH was administrated 2 h later. (n=6-8 per condition, *P<0.05; **P <0.01. *vs*. vehicle control, with ANOVAs followed by Bonferroni/Dunn *post-hoc* test). Data are presented as mean ± SEM.

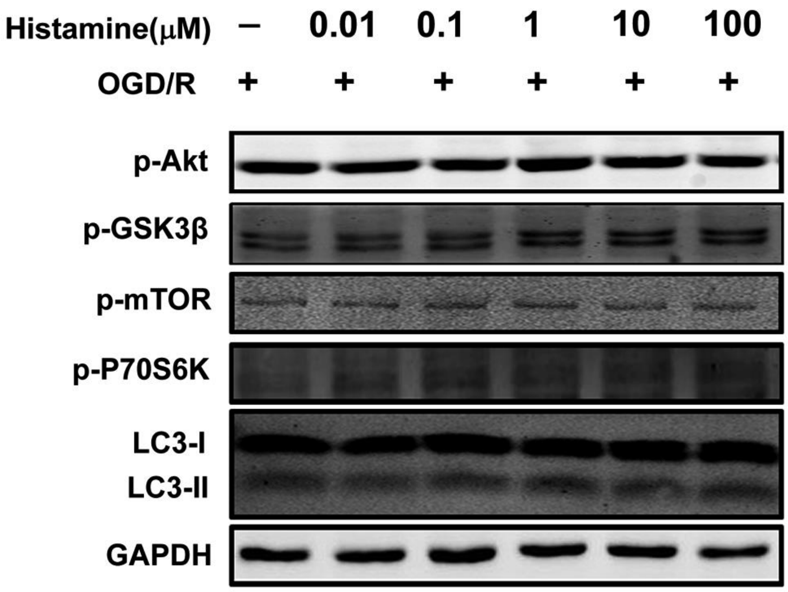

Supplementary Fig. 7 Histamine has no effects on autophagy activation. Primary cultured neurons were treated with indicated concentrations of histamine, and the protein level of p-Akt, p-GSK3β, p-mTOR, LC3 and GAPDH were examined by western blot. Full-size blots are shown in Supplementary Fig. 18.

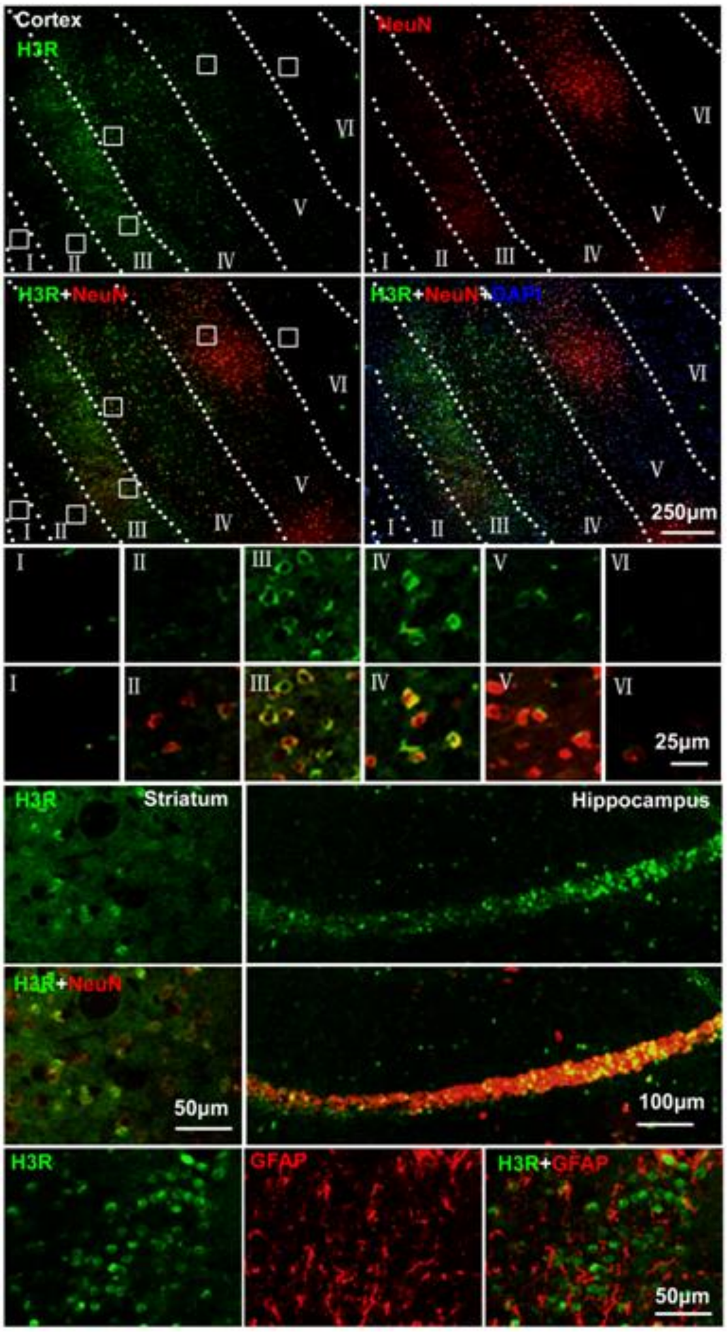

Supplementary Fig. 8 H3R localization in mouse brain revealed by immnohistochemistry. The 8-12 weeks old male C57BL/6 mouse brain sections were co-immunostained with H3R and neuronal marker NeuN and astrocytic marker GFAP. The localization of H3R in indicated cortical layers, hippocampus and striatum was observed by confocal microscopy. DAPI was used to label cell nucleus. The scale bars were labbled in indicated images.

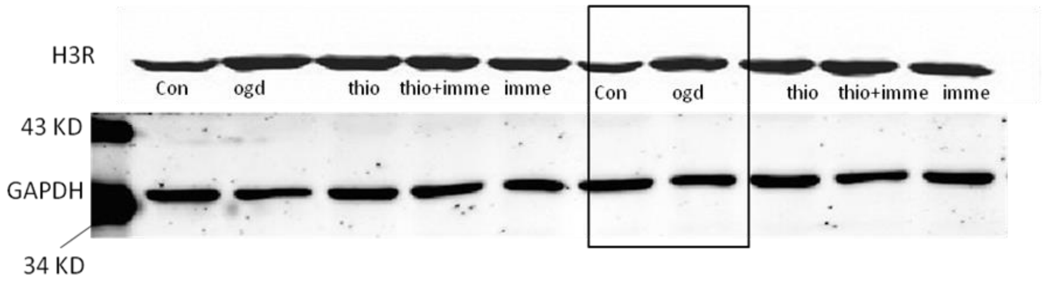

Full unedited gel for Figure 1a

Supplementary Fig. 9 Unedited full-size blots of Fig. 1a

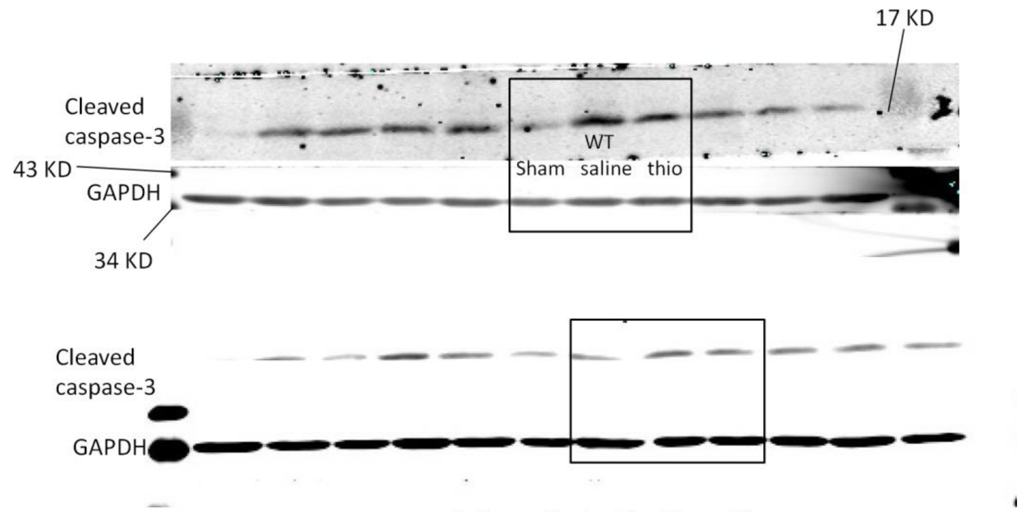

Full unedited gel for Figure 2b

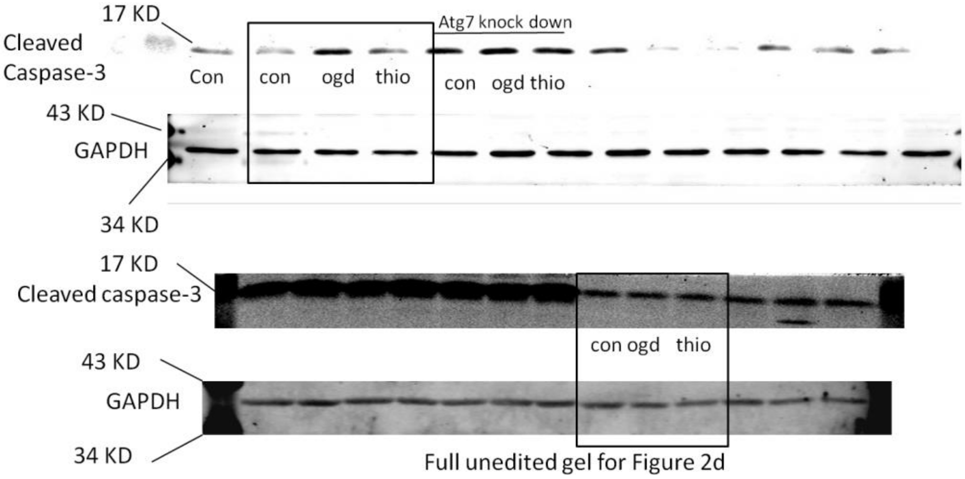

Full unedited gel for Figure 2d

Supplementary Fig. 10 Unedited full-size blots of Fig. 2

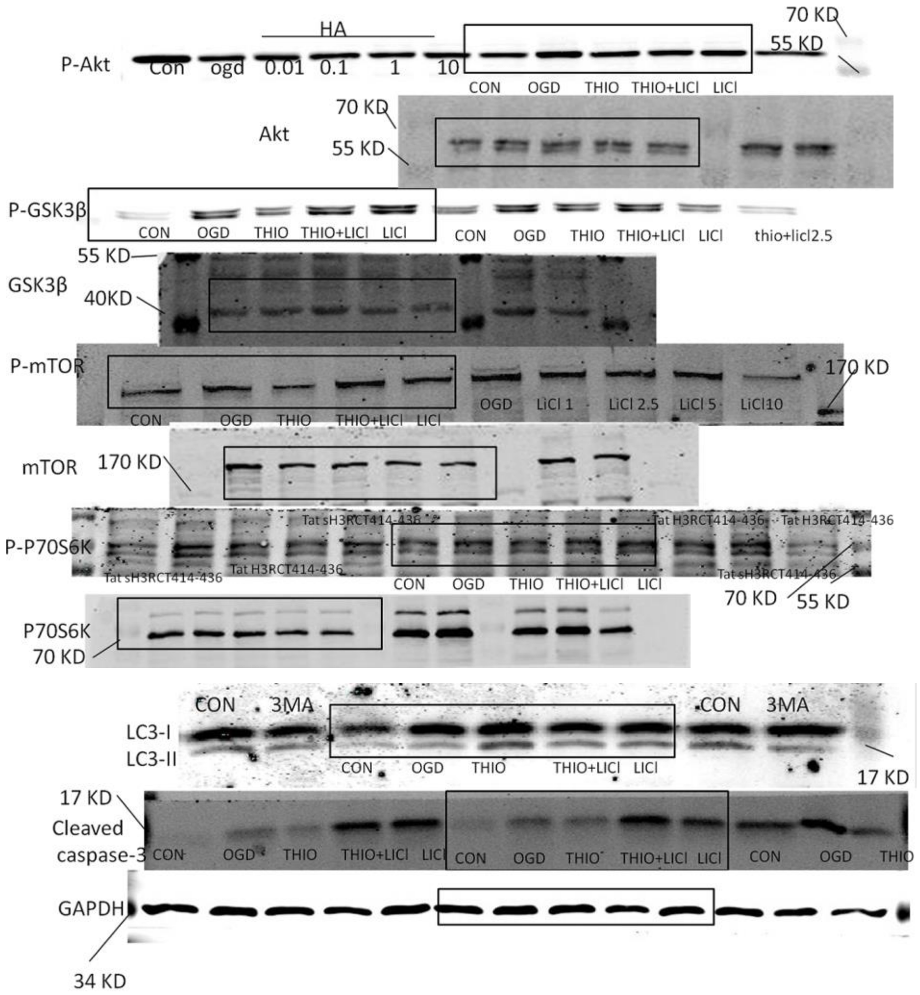

Full unedited gel for Figure 4a

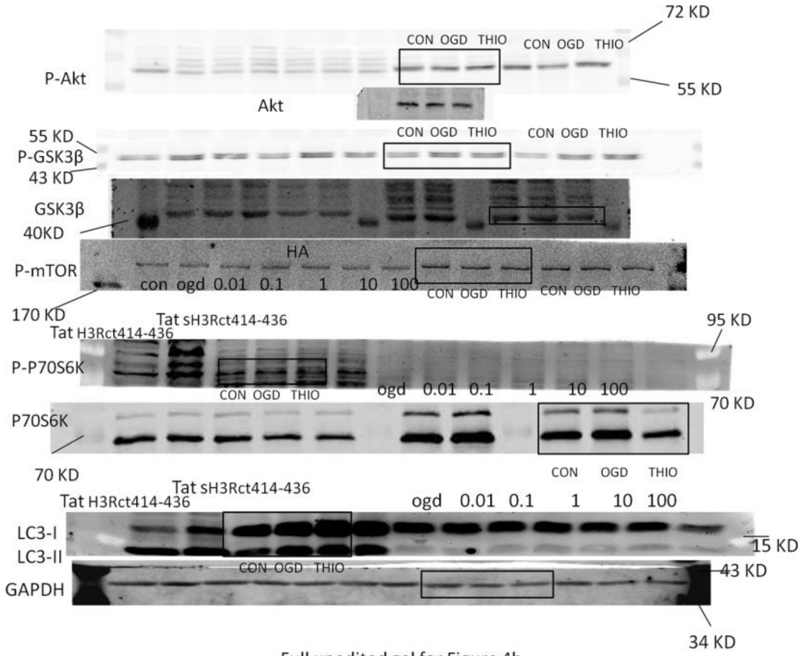

Full unedited gel for Figure 4b

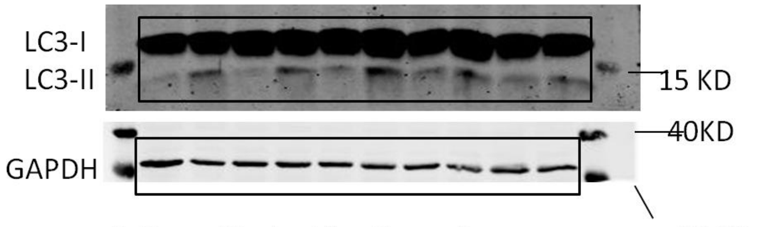

Full unedited gel for Figure 4e

Supplementary Fig. 11 Unedited full-size blots of Fig. 4

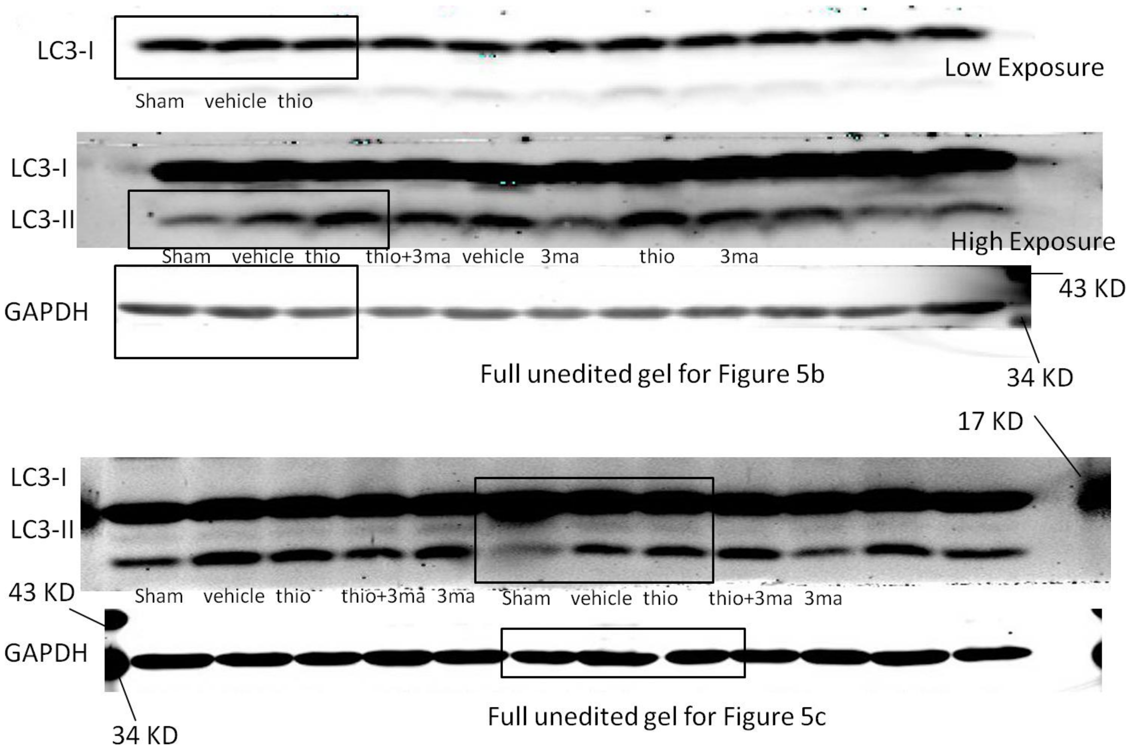

Supplementary Fig. 12 Unedited full-size blots of Fig. 5

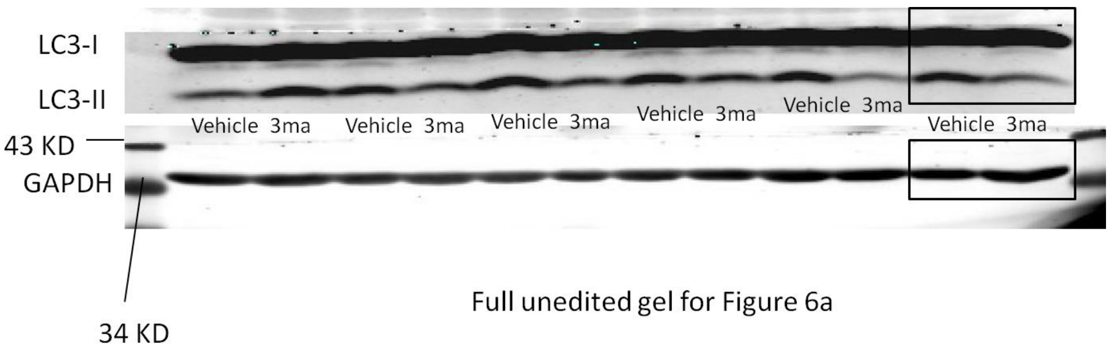

Supplementary Fig. 13 Unedited full-size blots of Fig. 6

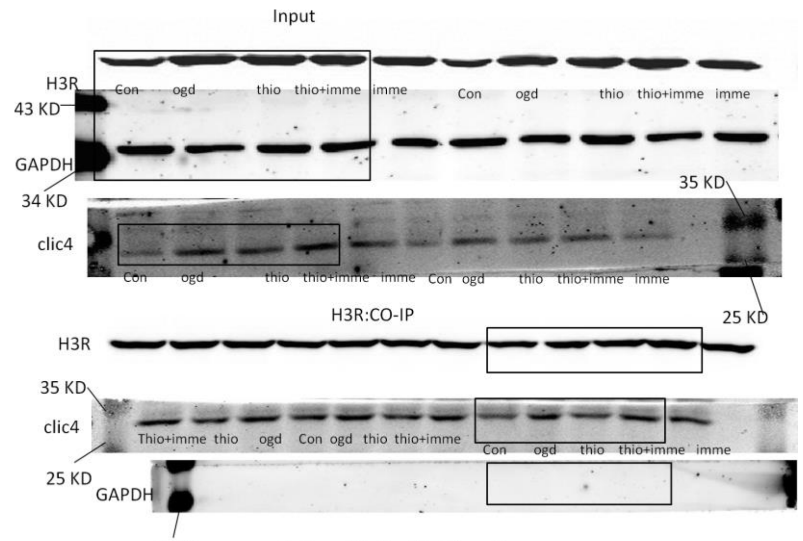

Full unedited gel for Figure 7a

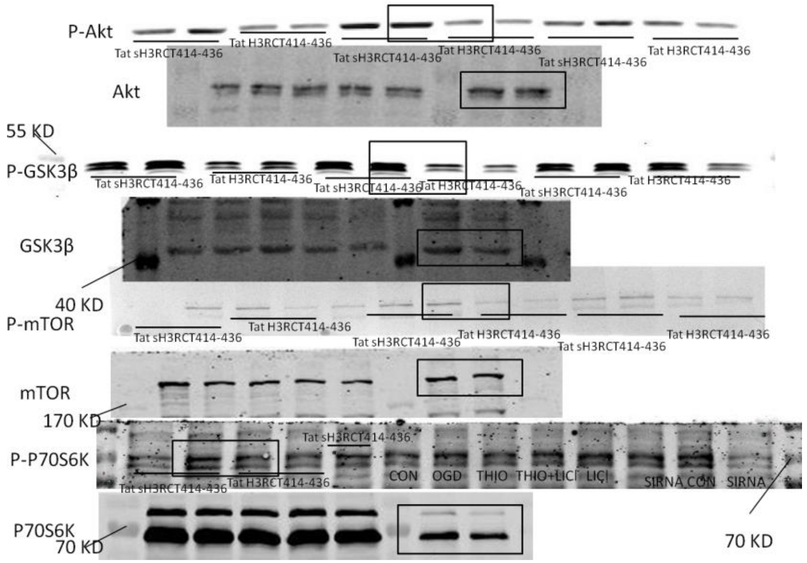

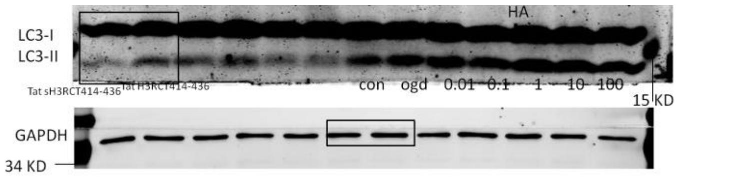

Full unedited gel for Figure 7c

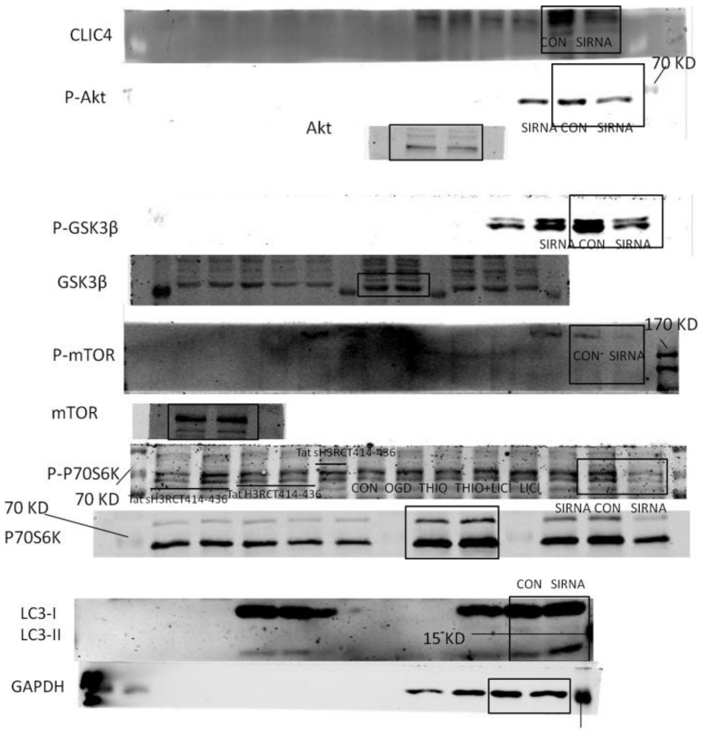

Full unedited gel for Figure 7e

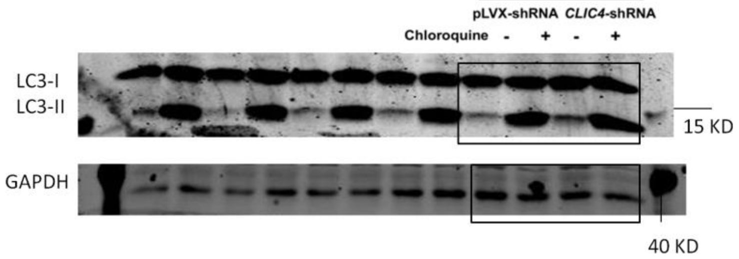

Supplementary Fig. 14 Unedited full-size blots of Fig. 7

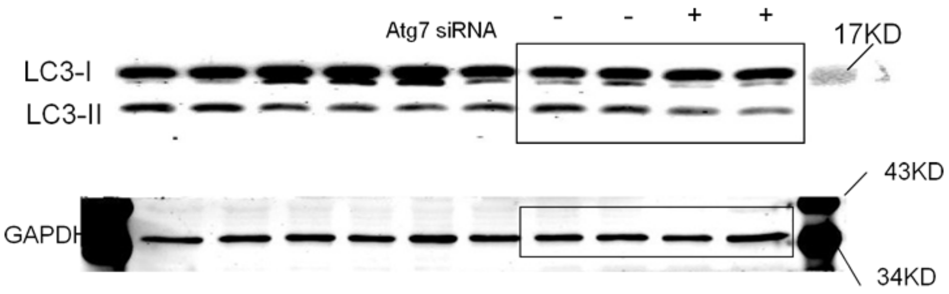

Full unedited blots for Supplementary figure 3

Supplementary Fig. 15 Unedited full-size blots of Supplementary figure 3.

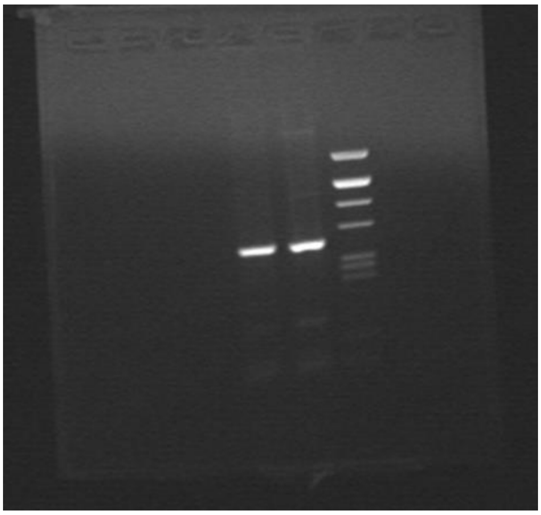

Supplementary Fig. 16 Unedited full-size gel of Supplementary figure 4.

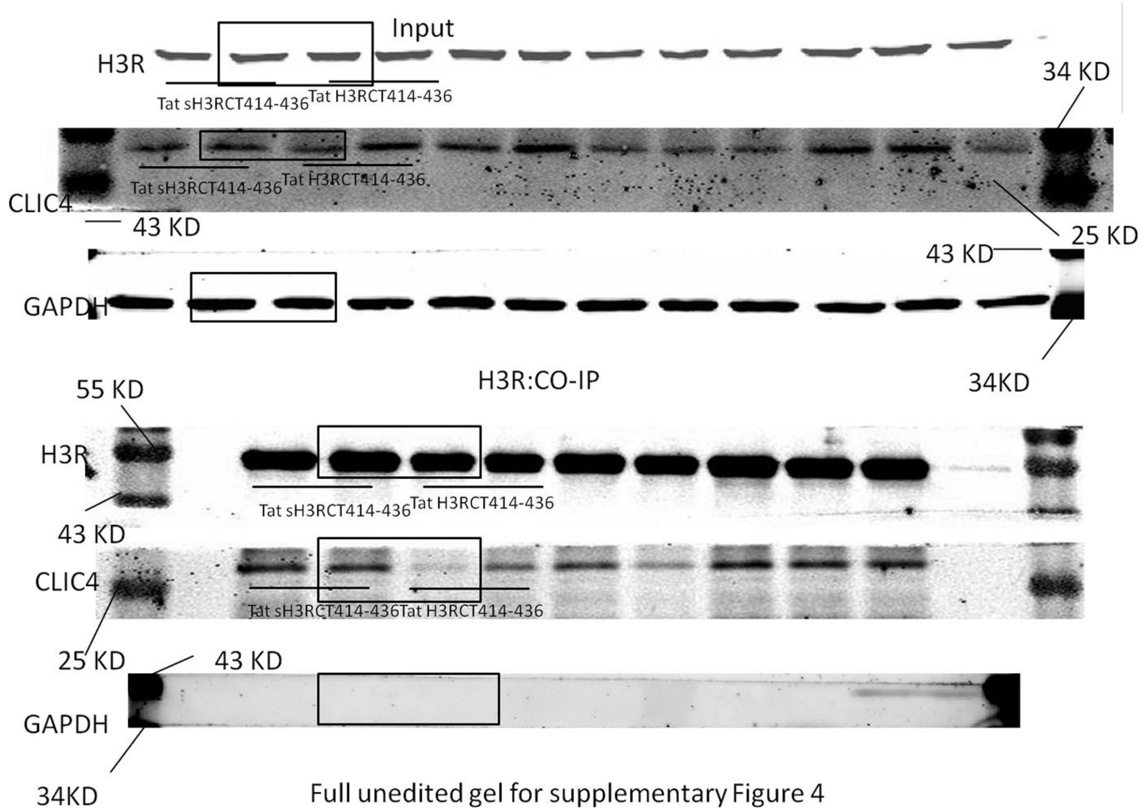

Supplementary Fig. 17 Unedited full-size blots of Supplementary figure 5.

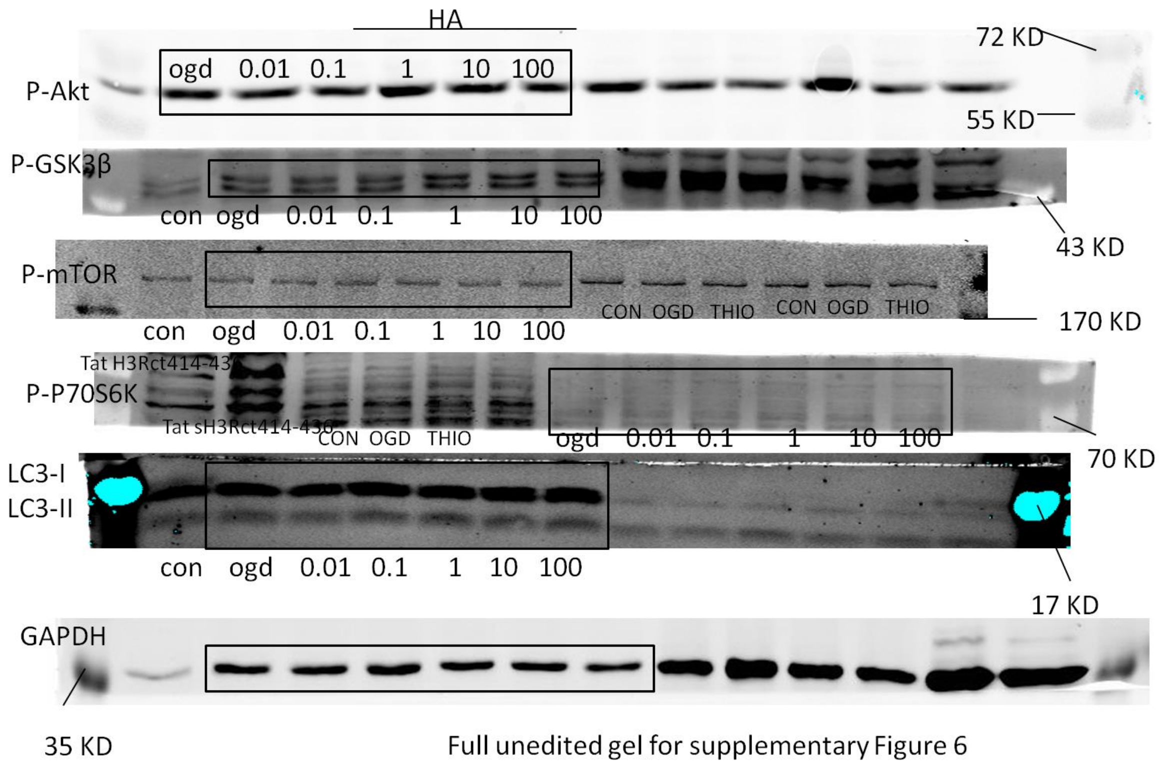

Supplementary Fig. 18 Unedited full-size blots of Supplementary figure 7.
